# Lineage-specific innovations expand mitochondrial proteomes in kinetoplastids

**DOI:** 10.1016/j.molcel.2026.08.032

**Published:** 2026-10-01

**Authors:** Andres F. Vacas, Md Solayman, Sarah E. Calvo, Khanh Nguyen, Cameron Genxuan Lian, Keith D. Rivera, Michael Z. Chen, MitoCarta Tree of Life Consortium, Namrata D. Udeshi, Vamsi K. Mootha, Ruslan Aphasizhev, Inna Aphasizheva

**Affiliations:** 1 Department of Molecular and Cell Biology, Boston University Medical Campus, Boston, MA 02118, USA; 2 Department of Molecular Biology, Massachusetts General Hospital, Boston, MA 02114, USA; 3 Department of Systems Biology, Harvard Medical School, Boston, MA 02115, USA; 4 Broad Institute of MIT and Harvard, Cambridge, MA 02142, USA; 5 Howard Hughes Medical Institute, Massachusetts General Hospital, Boston, MA 02114, USA; 6 These authors contributed equally; 7 Lead contact

**Keywords:** Kinetoplastids, *Trypanosoma*, *Leishmania*, mitochondria, comparative proteomics, protein correlation profiling (PCP), DIA-mass spectrometry, MitoCarta

## Abstract

*Trypanosoma brucei* and *Leishmania tarentolae* are model kinetoplastids whose mitochondria contain a unique DNA network, kinetoplast DNA (kDNA), and exhibit specialized features, including developmental remodeling and RNA editing. By integrating mass spectrometry–based protein correlation profiling with published datasets, we assembled inventories of 1,666 mitochondrial proteins in *T. brucei and* 1,594 in *L. tarentolae*. These include 124 polypeptides not previously recognized as mitochondrial. Despite their phylogenetic distance, trypanosomes and leishmanias maintain highly conserved mitoproteomes, including about 700 components with no detectable homologs in other eukaryotes. Comparison of *T. brucei* insect and mammalian stages revealed similar mitoproteome compositions but globally reduced mitochondrial protein abundance in the latter, except for components of calcium homeostasis and the alternative oxidase pathway. We also detected ten kDNA-encoded proteins, including products of pan-edited transcripts. These inventories establish a framework for functional analysis of previously uncharacterized mitochondrial factors and illuminate innovations that have shaped kinetoplastid evolution.

## INTRODUCTION

Kinetoplastids are unicellular flagellated eukaryotes, many of which cause vector-borne parasitic diseases of humans and animals, imposing a substantial public health and economic burden.^1^ This group includes *Trypanosoma brucei*, the causative agent of African trypanosomiasis (sleeping sickness); *Trypanosoma cruzi*, responsible for Chagas disease in the Americas; and several *Leishmania* species associated with cutaneous, mucocutaneous, and visceral leishmaniasis. Current treatments, such as pentavalent antimonials, amphotericin B, and miltefosine for leishmaniasis, and benznidazole, nifurtimox, suramin, and eflornithine for trypanosomiases, are limited by toxicity, inconsistent efficacy, and emerging drug resistance.^2^ Although the oral drug fexinidazole represents a major recent advance in the treatment of human African trypanosomiasis,^3^ the potential for resistance is unclear, and overall therapeutic options remain limited.

The mitochondrial genome of kinetoplastids, comprising maxicircles and minicircles collectively known as kinetoplast DNA (kDNA), is among their most distinctive cellular features. In *Trypanosoma* and *Leishmania*, maxicircles are interlocked with minicircles into a single catenated network compacted by histone-like proteins. Maxicircles correspond to mitochondrial genomes of other eukaryotes and encode 9S and 12S rRNAs, two ribosomal proteins, subunits of respiratory complexes, and predicted proteins of unknown function. Unlike most mitochondrial genomes, kDNA lacks tRNA genes,^4^ and transcripts from 12 maxicircle cryptogenes undergo U-insertion/deletion editing.^5^ In this unique process, kDNA-encoded guide RNAs (gRNAs) direct nuclear-encoded factors to restore protein-coding sequences in cryptogene transcripts,^6^ which subsequently undergo 3′ modification^7,8^ and translation by highly proteinaceous ribosomes.^9,10^ Specialized complexes also mediate replication and segregation of the kDNA network, contributing to mitochondrial proteome expansion through lineage-specific innovations.^11–15^

Kinetoplastid parasites adapt to diverse environments throughout their life cycles. In *T. brucei*, proliferative stages include the procyclic form in the tsetse fly midgut, where oxidative metabolism predominates, and the long slender bloodstream form in mammals, where ATP production relies primarily on glycolysis compartmentalized within peroxisome-like organelles termed glycosomes.^16^ *Leishmania* species likewise transition between extracellular promastigotes in sandfly vectors and intracellular amastigotes that survive within acidic, nutrient-limited macrophage lysosomes.^17^ These developmental transitions are accompanied by extensive remodeling of mitochondrial size, morphology, and metabolic and biosynthetic pathways; however, the corresponding mitoproteome dynamics remain poorly characterized.

In *T. brucei*, the mitoproteome has been extensively studied using a variety of “omics” approaches, including proteomics of purified mitochondria (PPM),^14,15,18^ proteomics of isolated mitochondrial complexes;^6,19^ hyperplexed localization of organelle proteins by isotope tagging (HyperLOPIT);^12,20^ genome-wide fluorescent tagging (TrypTag);^11,13^ and genetic perturbation of protein import machinery.^21^ Proteomic approaches, however, are limited by technical factors and the broad dynamic range of mitochondrial protein abundance, which together complicate discrimination of abundant contaminants from bona fide mitochondrial proteins and reduce sensitivity by limiting sampling depth. For example, in PPM, density-gradient purification enriches kDNA-containing mitoplasts, which form when the single tubular mitochondrion ruptures during cell lysis, thereby excluding a substantial fraction of organelle volume. HyperLOPIT combines MS-based proteomics, isobaric multiplexing, and machine learning to assign mitochondrial localization across coarse gradient fractions. In both approaches, limited gradient resolution and the dynamic range of data-dependent mass spectrometry reduce sensitivity and precision.

The TrypTag project achieved an 89% success rate in fluorescent tagging of 8,721 predicted protein-coding genes, excluding variant surface glycoproteins (VSGs) and duplicates. Of these, 1,050 C-terminally tagged proteins, hereafter referred to as the TrypTag-C dataset, were annotated as mitochondrial.^11^ However, many exhibited imprecise cellular localization due to low signal-to-noise ratios or artifactual sub-mitochondrial distribution caused by mNeonGreen’s intrinsic affinity for the kinetoplast nucleoid.^13^

In contrast, *Leishmania* mitoproteomes remain understudied,^22,23^ despite the increasing global burden of leishmaniasis. Here, we extend mitoproteome analysis to *L. tarentolae* and integrate methodological advances with cross-species comparisons and targeted analyses to establish a framework for functional studies within kinetoplastids and across eukaryotes.

As part of the MitoCarta Tree of Life (Mito-TOL) project, we analyzed mitoproteomes from procyclic (TbPCF) and bloodstream (TbBSF) forms of *T. brucei* Lister 427 and from *L. tarentolae* promastigotes (LtPMF, UC strain). By integrating affinity-, proteolytic-, and buoyancy-based purification strategies with data-independent acquisition (DIA) mass spectrometry and protein correlation profiling (PCP), we expanded the known mitoproteomes by ~50%. We further characterized stage-specific variation in mitochondrial protein abundance across the *T. brucei* life cycle. By incorporating published datasets, the resulting MitoCarta resources advance understanding of mitochondrial contributions to parasite adaptation, uncover numerous previously uncharacterized protein families, and provide insight into both lineage-specific innovations and conserved mitochondrial features tracing back to the last eukaryotic common ancestor (LECA).

## RESULTS

### Protein correlation profiling defines the *Trypanosoma brucei* mitochondrial proteome

To assess the depth and accuracy of existing *T. brucei* mitoproteome annotations, we benchmarked published datasets derived from proteomics of purified mitochondria (PPM),^14^ HyperLOPIT,^12^ genome-wide C-terminal fluorescent tagging (TrypTag-C),^11^ genetic perturbation of the protein import machinery (Importome),^21^ and mitochondrial sub-fractionation studies, including the mitochondrial outer membrane (MOM)^24^ and respiratory complexes (Respiratome)^18^ repertoires (Figure 1A; Table S1).

The pioneering PPM analysis by Panigrahi et al. (2009)^14^ achieved 67% sensitivity with a corrected false discovery rate (cFDR) of 4%, identifying 887 mitoproteins in TbPCF. Subsequent methodological advances achieved comparable specificity while increasing proteome depth. For example, Moloney et al. (2024)^12^ identified 1,090 proteins with a cFDR of 3% using HyperLOPIT. The genome-wide C-terminal fluorescent tagging^11^ also achieved a low cFDR (2%).

However, these datasets are only partially concordant, yielding an aggregate of 1,794 proteins, of which ~13% are predicted false positives (Figure 1A). This analysis highlights the limitations and biases of previous approaches and underscores the need for a high-confidence reference mitoproteome.

To address these limitations, we developed two complementary purification strategies: one combining Proteinase K (PK) treatment with density-gradient (DG) centrifugation, and another adding an immunoprecipitation (IP) step (Figure S1A). To enable IP, the mitochondrial outer membrane protein POMP10^25^ was C-terminally tagged with enhanced green fluorescent protein (eGFP) fused to a Twin-Strep (TS) tag^26^ (Figures 1B and 1C). In this workflow, isotonic cell lysis by nitrogen cavitation was followed by either IP with subsequent PK treatment (Figure 1D) or PK treatment prior to DG separation. These approaches yielded progressively enriched preparations providing benchmarks for quantitative assessment of mitochondrial protein abundance at each step (Figures 1E, S1B, and S1C).

Mass spectrometry (MS)^27^ confirmed enrichment of mitoproteins across purification steps (Figures 1F, S1D and S1E; Table S2). In whole-cell lysates, we identified 7,606 proteins (6,948 protein groups), representing 79% of the *T. brucei* proteome. Subcellular assignments from the HyperLOPIT showed a stepwise increase in mitochondrially localized proteins, accompanied by depletion of proteins from other organelles, across successive purification stages (Figure 1G).

We next applied protein correlation profiling (PCP)^28^ using the conserved mitochondrial markers TOM40, Hspd1, and IscU as a reference set adopted across the Mito-TOL project. Proteins exhibiting a PCP (Pearson correlation) score ≥ 0.6 were classified as mitochondrial and collectively designated as the TbMitoViaPCP dataset (Figure 1H; Table S3).

Benchmarking the 1,577 TbMitoViaPCP proteins against manually curated “Gold+,” “Possible mito,” and “Other” reference sets demonstrated 90% sensitivity with a 7% cFDR (Figure 1I). The IP+PK+DG workflow yielded modest performance gains over PK+DG (Figures S1F and S1G; Table S4), and both pipelines substantially outperformed published datasets (Figure 1J).

Among the 1,577 TbMitoViaPCP proteins, 842 overlapped with the 887 identified by PPM,^14^ 953 overlapped with the 1,057 proteins assigned to mitochondria in Tryp-Tag-C,^11^ and 1,060 with the HyperLOPIT dataset^12^ (Figure 1K; Table S1). The TbMitoViaPCP proteins span nearly eight orders of magnitude in abundance, underscoring the depth of coverage achieved and enabling the identification of 124 previously unrecognized mitoproteins (Figure 1L). Overall, the sensitivity and accuracy of TbMitoViaPCP, together with its strong agreement with prior datasets, support a substantial expansion of the mitochondrial proteome in kinetoplastids.

### Stage-dependent remodeling of the *T. brucei* mitoproteome

During the vector-to-host transition, the mitochondrion of *T. brucei* undergoes profound morphological and functional remodeling,^16,29^ switching from ATP synthesis via oxidative phosphorylation (OXPHOS) to ATP hydrolysis to maintain the membrane potential.^30^ To assess whether the mitochondrial proteomes differ between the respective stages, we analyzed TbBSF using a streamlined PK+DG workflow (Figures 2A, S2A, and S2B).

Of the 1,577 proteins in the TbPCF MitoViaPCP dataset, 1,457 (92%) were also enriched in TbBSF mitochondria, showing PCP scores > 0.3 (Figure 2B; Table S5). Benchmark analysis indicated higher contamination in TbBSF preparations, with cFDR increasing from 7% in PCF to 26% in BSF (Figure 2C; Table S4). The contaminants were derived mainly from secretory, endoplasmic reticulum, cytosolic, and acidocalcisomal proteins (Figures S2C and S2D).

Comparison of the TbBSF dataset (1,640 proteins with PCP scores > 0.9, with estimated ~426 false positives) with TbMitoViaPCP (1,577 proteins, with estimated ~110 false positives) and the HyperLOPIT dataset^12^ (Figure 2D) showed extensive overlap between TbBSF and TbMitoViaPCP (1,273 proteins). Of the remaining TbBSF proteins, 361 were unique to the TbBSF PCP dataset, whereas only six additional proteins overlapped with HyperLOPIT but not with TbMitoViaPCP. Given the higher contamination in TbBSF (~426 false positives), nearly all 361 TbBSF-specific proteins are unlikely to be mitochondrial, particularly because they exhibited non-mitochondrial patterns in TbMitoViaPCP (Figure 2B). Together, our results indicate that the TbPCF and TbBSF mitoproteomes are qualitatively similar. TbBSF-specific mitoproteins, if present, are rare, and the apparent absence of some TbMitoViaPCP proteins in TbBSF likely reflects their low abundance (Figure S2E and S2F).

Having established that mitoproteome composition remains largely similar between developmental forms, we next examined quantitative differences in whole-cell lysates from TbPCF and TbBSF in a triplicate proteomics experiment (Figures 2E and 2F; Table S5). Whereas the number of detected TbMitoViaPCP protein groups was comparable between TbPCF (1,462) and TbBSF (1,384) (Figure 2E), their relative abundances differed markedly: mitoproteins accounted for ~16% of total protein intensity in TbPCF but only ~8% in TbBSF (Figure 2F). Consistent with this, mitochondrial and non-mitochondrial protein abundances were highly correlated between stages (mean values from three whole-cell lysate replicates). Nevertheless, TbMitoViaPCP proteins displayed an overall shift toward higher abundance in TbPCF (Figure 2G). Notably, components of OXPHOS complexes III and IV deviated strongly from the overall distribution, showing depletion in TbBSF.

Pathway-level analysis confirmed that nearly all mitochondrial pathways are quantitatively downregulated in TbBSF, underscoring a global reduction in mitochondrial metabolic and respiratory capacity (Figure 2H). Notably, OXPHOS complexes III and IV declined disproportionately, suggesting that they are effectively dismantled in TbBSF. In line with this global trend, most mitoproteins were less abundant in TbBSF, while some were significantly upregulated (Figure 2I). Those included trypanosome alternative oxidase (TAO; Tb927.10.7090), glycerol-3-phosphate dehydrogenase (mtG3PDH; Tb927.11.7380), alanine transaminase (ALAT; Tb927.1.3950), the mitochondrial calcium uniporter (MCU; Tb927.11.1350), L-2-hydroxyglutarate dehydrogenase (L2HGDH; Tb927.10.9360), and Tb927.9.8950 (CAAX prenyl protease 1), in agreement with previous studies.^31–33^

Together, these results indicate that the principal differences between insect and bloodstream stages reflect primarily protein abundance shifts commensurate with pathway activity, whereas the overall composition of the mitochondrial proteome remains largely similar.

### Defining the *L. tarentolae* mitoproteome by protein correlation profiling

To investigate mitoproteome differences between distantly related kinetoplastids occupying distinct ecological niches, we next applied our mitochondrial enrichment and correlation profiling workflow to promastigotes of *L. tarentolae* (LtPMF, UC strain), a parasite of geckos^34^ transmitted by sandflies.

We found that immunoaffinity isolation did not substantially improve mitochondrial enrichment (Figure S3A and S3E). In contrast, adjusting the OptiPrep concentration to 10–30% in the PK+DG workflow (Figure 3A) yielded preparations with the highest precision and sensitivity (Figures S3C and S3D). Transmission electron microscopy confirmed progressive enrichment of mitochondrial vesicles across purification steps (Figure 3B). Marker mitoproteins RESC1/2,^35^ ATOM40,^25^ and 3-methylcrotonyl-CoA carboxylase^36^ (MCC) increased steadily, whereas cytosolic acetyl-CoA carboxylase (ACC) and β-tubulin declined below detection (Figure S3B). Mass spectrometry showed parallel increases in mitochondrial protein numbers and total signal intensity (Figure 3C; Table S6), accompanied by depletion of contaminants (Figure 3D).

As above, using Tom40, Hspd1, and IscU as mitochondrial reference markers, PCP identified 1,531 mitoproteins in LtPMF (LtMitoViaPCP), with 93% sensitivity and a 7% cFDR (Figures 3E and 3F; Tables S4 and S7). This represents an approximately threefold expansion relative to previously reported *Leishmania* mitoproteomes.^23^

### MitoCarta inventories of *T. brucei* and *L. tarentolae*

Although *Trypanosoma* and *Leishmania* diverged approximately 120 million years ago (MYA),^37^ their MitoViaPCP datasets of nuclear-encoded proteins largely overlapped and displayed highly concordant sensitivity and cFDR values. Ortholog-matched comparisons showed 92–93% similarity between the two species, with only 122 and 112 MitoViaPCP proteins unique to *T. brucei* and *L. tarentolae*, respectively (Figure 4A; Tables S3 and S7). Given the 7% cFDR, these proteins likely comprise a mixture of false positives and genuine lineage-specific components.

To validate the MitoViaPCP inventories by microscopy, we modified selected proteins with a C-terminal CTS tag.^38^ This compact tag does not typically interfere with protein function or sub-mitochondrial localization^39^ and enables affinity purification and immunofluorescence microscopy. Four protein categories (Figure 4B) were selected for validation by confocal microscopy (Figures 4C–4F). Category I includes candidates assigned to both MitoViaPCP inventories but designated as non-mitochondrial in the TrypTag-C dataset; these were tested in TbPCF. Category II comprises proteins classified as non-mitochondrial in both MitoViaPCP datasets but annotated as mitochondrial in the TrypTag-C; these were tested in TbPCF. Category III comprises *L. tarentolae* MitoViaPCP proteins lacking detectable *T. brucei* orthologs; these were tested in LtPMF. Category IV contains proteins identified as mitochondrial in only one MitoViaPCP inventory and annotated as mitochondrial or non-mitochondrial in TrypTag-C; these were also tested in LtPMF.

The validation outcomes for Categories I–III were consistent with the low cFDR estimates for both MitoViaPCP inventories. Immunofluorescence confirmed mitochondrial localization for all four Category I candidates in TbPCF, including proteins annotated as non-mitochondrial in the TrypTag-C (Figure 4C). KRET1 (Tb927.7.3950) was evaluated using monoclonal antibodies, as previous work showed that C-terminal tagging does not preserve its mitochondrial localization.^40^

Category II represents the converse case, in which proteins annotated as mitochondrial by TrypTag-C were not supported by MitoViaPCP (Figure 4D), underscoring the value of orthogonal approaches for assigning mitochondrial localization. In Category III, all five candidates localized to the *L. tarentolae* mitochondrion (Figure 4E), indicating that the comparative approach successfully identified bona fide genus-specific mitoproteins.

By contrast, Category IV yielded mixed outcomes (Figure 4F). Three proteins classified as mitochondrial in TbMitoViaPCP and TrypTag-C were confirmed as non-mitochondrial by immunofluorescence analysis of their *L. tarentolae* orthologs, consistent with the LtMitoViaPCP assignments. Conversely, three proteins classified as mitochondrial in LtMitoViaPCP localized to the mitochondrion in *L. tarentolae*, despite their *T. brucei* orthologs being classified as non-mitochondrial in both TbMitoViaPCP and TrypTag-C; one of these proteins exhibited dual localization. Only a single protein showed localization inconsistent with the corresponding MitoViaPCP assignment within the same organism.

These findings suggest that Category IV includes a mixture of species-specific mitoproteins, dual-localized proteins, and false-positive assignments. For example, LtaP33.0490 exhibited dual nuclear and mitochondrial localization, with a stronger nuclear signal, consistent with both datasets. Overall, these results indicate that comparative cross-species protein-correlation profiling, combined with orthogonal localization datasets and targeted microscopy validation, improves the interpretation of species-specific and ambiguously localized proteins and enhances the accuracy and depth of mitochondrial inventories.

To assemble comprehensive mitoproteomes, we integrated the MitoViaPCP datasets with proteins validated by the TrypTag project that are supported by PCP evidence (PCP > 0; 995 in *T. brucei* and 913 orthologs in *L. tarentolae*), together with a manually curated, literature-based “Gold+” set of mitochondrial proteins (Tables S3 and S7). The latter includes microscopy-validated markers, components of purified mitochondrial complexes, and protein chains resolved in cryo-EM structures (546 in *T. brucei* and 502 orthologs in *L. tarentolae*). The resulting reference inventories, termed TbMitoCarta and LtMitoCarta, comprise 1,666 and 1,594 proteins, respectively (Figure 4F; Tables S3 and S7).

### Dual-targeted proteins

Whereas protein correlation profiling (PCP) identifies mitoproteins with high sensitivity and precision, it can miss dual-targeted proteins, such as aminoacyl-tRNA synthetases (aaRSs) that localize to both the cytosol and mitochondrion. Consistent with their dual localization, these proteins are generally not classified as mitochondrial by TrypTag^11^ or HyperLOPIT^12^ and typically lack targeting signals based on TargetP 2.0^41^ and DeepLoc 2.1^42^ predictions (Figure S4).

Dual-localized aaRSs displayed low PCP scores when whole-cell fractions were included but showed increased enrichment upon their exclusion, particularly in *T. brucei* (Figure S4). Although similar trends were observed in *L. tarentolae* (Figures S4A and S4C), the lower confidence of the corresponding dataset precluded rigorous identification of a dual-targeted candidate set. Based on this behavior, we defined “TbDualMito” proteins as those that show low scores in the full dataset (PCP < 0.60) but strong enrichment after excluding whole-cell fractions (PCP > 0.85).

For clarity, these proteins are not included in the TbMitoCarta or LtMitoCarta inventories, but are instead provided as companion resources. Using these criteria, we identified 64 TbDualMito genes, including all 18 dual-localized aaRSs and 46 additional candidates requiring further validation (Table S3D).

### Mass spectrometry detects proteins encoded by kDNA genes and cryptogenes

Notwithstanding the evolutionary divergence between *Trypanosoma* and *Leishmania* and the markedly different histories of the laboratory strains analyzed here, their nuclear-encoded mitoproteomes are highly concordant (Figure 4A). The opposite trend would be expected for kDNA-encoded proteins, as both *T. brucei*^43–45^ and *L. tarentolae*^46,47^ exhibit extensive streamlining of their mitochondrial genomes during prolonged cultivation.

Although maxicircles encoding rRNAs and proteins remain comparatively stable, minicircles that supply the gRNAs required for mRNA editing are highly malleable. When a protein becomes dispensable under culture conditions, for example, components of the respiratory complex I,^48^ the corresponding mRNAs are no longer edited because the minicircles encoding cognate gRNAs are rapidly lost during cell divisions. Experimental evidence for translation of mitochondrial mRNAs has remained limited to N-terminal Edman sequencing of cytochrome b^49^ and cytochrome oxidase subunit I (COXI)^50^ in LtPMF, and of ATP synthase subunit 6 (ATP6) in TbPCF,^51^ and to the identification of ribosomal proteins uS3m and uS12m, as well as ATP6, in cryo-EM structures of their respective complexes.^9,52^ Mass spectrometry detected the protein product of the mitochondrial unidentified reading frame 2 (MURF2)^53^ and subunits of Complex I in several trypanosomatid species.^54^

Our MS analysis across mitochondrial purification steps identified nine kDNA-encoded proteins in each organism. ND2 was found only in TbPCF and ND7 only in LtPMF, bringing the combined total to 10 of the 18 predicted kDNA-encoded proteins. Notably, nearly all these proteins were detected in both mitochondrial fractions and whole-cell lysates (Figure 5A; Table S8).

These proteins included subunits of respiratory complexes I (ND1, ND2, ND7), III (CYB), IV (COX1–3), and V (ATP6), as well as the mitoribosomal protein uS3m, and represent products of never-edited (ND1, ND2, COX1, uS3m), moderately edited (CYB, COX2), and pan-edited (ND7, COX3, ATP6) mRNAs. In addition, we identified the translation product of MURF2 mRNA, which derives from a functionally unassigned cryptogene and requires editing, in LtPMF and in both forms of *T. brucei*. We note that only uS3m and MURF2 were detected in TbBSF.

It appears that low abundance, rather than insolubility under extraction conditions, accounts for the absence of the predicted CR3 and CR4 polypeptides, which lack detectable homology to proteins outside Kinetoplastea, as well as the conserved ND3–5, ND8–9, and uS12m proteins.

Although kDNA-encoded proteins are generally more hydrophobic than their nuclear-encoded counterparts within the same complexes, there is no systematic hydrophobicity bias distinguishing detected from undetected kDNA-encoded proteins (Figure 5B). Instead, the missing proteins belong to complexes with the lowest overall abundance, such as complex I and the small mitoribosomal subunit. Consistent with this interpretation, the kDNA-encoded proteins are recovered at levels several orders of magnitude below those inferred for nuclear-encoded components of the same complexes (Figures 5C and 5D).

The comparatively low expression of Complex I observed in TbPCF and LtPMF (Figure 5C) is consistent with previous reports showing that, in cultured trypanosomatids, Complex I exhibits incomplete assembly and weak rotenone-sensitive NADH oxidation, resulting in only a minor contribution to mitochondrial electron transport chain flux relative to other respiratory complexes.^18,55,56^ Nevertheless, we detected 51 of the 52 annotated nuclear-encoded subunits of *T. brucei* Complex I, together with their 48 orthologs in *L. tarentolae*, indicating that the comparatively low abundance of Complex I is unlikely to result from widespread loss of its constituent subunits. The single undetected subunit, Tb927.3.5340 (NDUFS5 homolog)^54^ is assigned as cytoplasmic in HyperLOPIT and TrypTag datasets, suggesting that it is likely misannotated rather than absent.

### Concordant expression of mitoproteins in insect stages of *T. brucei* and *L. tarentolae*

The insect-stage forms of *T. brucei* and *L. tarentolae* proliferate under distinct nutrient conditions but maintain active oxidative phosphorylation (OXPHOS) at the same cultivation temperature of 27 °C, whereas the bloodstream form of *T. brucei* grows at 37 °C and relies primarily on glycolysis.

To assess accompanying mitoproteome variation, we analyzed triplicate mass spectrometry quantifications of whole-cell lysates from TbPCF, TbBSF, and LtPMF. Comparable numbers of total and MitoCarta proteins were detected across all three proteomes. The insect stages of both organisms exhibited similar fractions of total MS intensity attributable to MitoCarta proteins and comparable proportional distributions across major mitochondrial pathways. However, LtPMF displayed a lower relative abundance of metabolic enzymes and a higher abundance of OXPHOS components (Figure 6A).

Next, we restricted our analysis to *L. tarentolae* and *T. brucei* MitoCarta orthologs detected in all replicates (n = 1,329) and normalized their protein intensities to compare relative expression between the two species (Table S9). Overall, protein abundance profiles were highly concordant, with a Pearson correlation coefficient of 0.8 (Figure 6B). Using gene set enrichment analysis (GSEA)^57,58^ with manually curated mitochondrial pathway annotations, we identified three complexes with significant interspecies differences (adjusted q < 0.03). OXPHOS complexes II and V were more abundant in LtPMF, whereas components of the RNA editing machinery,^6^ driven primarily by enrichment of the RNA editing catalytic complex (RECC), were more abundant in TbPCF (Figure 6B).

These differences align with the substantially higher oxygen consumption by *L. tarentolae*^59^ and more extensive U-insertion/deletion RNA editing in *T. brucei.*^60^ Overall, mitoprotein expression profiles diverge more between the insect and bloodstream stages of *T. brucei* than between the insect stages of these two species (Figure 6A).

### Kinetoplastid mitochondrion diverged through lineage-specific innovations

*T. brucei* and *L. tarentolae* MitoCartas, together with other Mito-TOL inventories, enable comparison of mitoproteome evolution across eukaryotes. Members of the class Kinetoplastea, *Trypanosoma,* and *Leishmania* diverged approximately 120 MYA^37,61,62^ within the Discoba clade, which itself separated from other eukaryotic lineages more than one billion years ago^61^ (Figure 7A). As expected,^63^ mitoproteins show greater conservation between *T. brucei* and *L. tarentolae* (95% shared orthology) than do their cellular proteomes (~80%) (Figure 7B).

To assess mitoproteome conservation, we leveraged orthologous groups (OGs, protein families) defined by the Mito-TOL project for 203 eukaryotic species and 34 prokaryotic clades (Table S10).^64^ Among the 1,503 OGs represented in *T. brucei* and *L. tarentolae* MitoCartas, 43% show detectable homology to prokaryotic proteins, 11% span multiple eukaryotic clades, and the remaining 46% lack detectable homologs outside of Discoba, suggesting lineage-specific innovation (Figure 7C). Evolutionary rate modeling based on sequence divergence within Discoba indicated that slowly evolving (powered) OGs likely represent genuine lineage-specific proteins, whereas short or rapidly evolving (underpowered) OGs are too divergent to reliably trace homology across eukaryotes (Figure 7C).

Both the number and fraction of lineage-specific protein families are far greater in kinetoplastids than in human, yeast, or *Arabidopsis* mitoproteomes, highlighting extensive mitochondrial diversification (Figure 7D). Functional annotation and pathway analysis revealed strong conservation of core metabolic enzymes, including those involved in acetate, amino acid, carbohydrate, lipid, and mevalonate metabolism. In contrast, proteins mediating mitochondrial gene expression, such as kDNA maintenance, ribosome biogenesis, and RNA metabolism, exhibit pronounced lineage-specific patterns, reflecting the unique information storage and processing systems of kinetoplastid mitochondria. Respiratory complexes and protein import machinery also show a comparable degree of divergence (Figure 7E).

We next assessed the homology of kinetoplastid MitoCarta proteins to known mitochondrial proteins or domains associated with mitochondrial functions. Because the above OG analysis included proteins that are mitochondrial in Kinetoplastea but may localize differently in other eukaryotes, we searched for OGs whose members are mitochondrial in human, yeast, or *Arabidopsis*. This search identified only 479 mitochondrial protein families in these species (Figure 7F). Among the remaining 1,024 OGs without detectable mitochondrial homologs, 531 lack recognizable PFAM domains, Gene Ontology (GO) or pathway assignments, or annotation in the TriTrypDB resource^65^ (Figure 7F). Conversely, 98 distinct PFAM domains are mitochondrial-only in the kinetoplastid MitoCarta and not mitochondrial in any other Mito-TOL inventories, potentially reflecting uncharacterized mitochondrial functions or domains that have adopted new roles in mitochondria of these species (Table S10).

Although the mitoproteomes of *T. brucei* and *L. tarentolae* are highly concordant (Figure 7B), several pathways are absent from one species or the other (Figure 7G). First, multiple *Trypanosoma* species have lost cobalamin-associated mitochondrial enzymes present in humans and in *L. tarentolae*—MUT (*LtaP27.0290*), MMAA (*LtaP03.0560*), MMAB (*LtaP24.2400*), and MCEE (*LtaP26.0030*)—as well as the related cytosolic enzyme MTR (*LtaP07.0090*) (Figure 7G). These losses are consistent with prior observations that *T. brucei* and *T. cruzi* lack the cobalamin-dependent methionine synthesis pathway and instead rely on cobalamin-independent routes, whereas *Leishmania* retains both cobalamin-dependent and cobalamin-independent enzymes.^66^

Second, several *Leishmania* species no longer encode the GDP-fucose biosynthetic enzymes GMD and GMER, whose *T. brucei* homologs (*Tb927.10.15490* and *Tb927.11.13990*) localize to mitochondria,^9^ whereas the related fucosyltransferase FUT1 (*Tb927.9.3600*/*LtaP01.0100*) is mitochondrial in both *T. brucei* and *L. tarentolae*^67,68^ (Figure 7G; Table S10). These findings are consistent with the loss of de novo GDP-fucose synthesis in *Leishmania*, which relies exclusively on fucose salvage, whereas *T. brucei* retains the biosynthetic route and *T. cruzi* maintains both.^69^

Together, these analyses demonstrate that the mitoproteomes of *Trypanosoma* and *Leishmania* are broadly conserved yet have diverged from other eukaryotic lineages through extensive lineage-specific innovation.

## DISCUSSION

Within early-branching Discoba, the kinetoplastid genera *Trypanosoma* and *Leishmania* manifest both deep conservation and lineage-specific divergence, sharing ~80% of their cellular proteomes yet maintaining ~95% overlap in mitochondrial proteins. The discovery of over 1,000 Kinetoplastea-specific mitoproteins in this study reveals an extensive, largely unexplored gene space that underlies essential organellar functions and reflects evolutionary innovations that may have arisen through both adaptive and neutral processes.

Examples of molecular complexity accommodated by dramatic mitoproteome expansion include: (i) the kinetoplast nucleoid, a concatenated, protein-condensed DNA network;^70^ (ii) RNA editing machineries that generate translatable mRNAs^6,39^ through interactions between kDNA-encoded cryptogene transcripts and gRNAs; and (iii) large, protein-rich mitoribosomes^9,10^ that house the smallest known rRNAs,^71,72^ and others. These mitochondrial systems have been discussed in both adaptive and neutral evolutionary contexts,^73^ and our study provides a high-confidence resource for the structural investigation of their lineage-specific components.^74^

Our mitochondrial purification strategy combined several complementary modalities to address the limitations of prior approaches and minimize contamination from distinct sources. Finely calibrated nitrogen cavitation under isotonic conditions preserved outer-membrane integrity, while immunoprecipitation enabled recovery of mitochondrial vesicles regardless of kDNA content.^75^ Proteinase K treatment was optimized to remove cytoskeletal contaminants without compromising outer-membrane proteins, and subsequent separation on an OptiPrep gradient efficiently eliminated glycosomal impurities. Transmission electron microscopy of the enriched fractions confirmed the presence of intact mitochondrial vesicles and visualized condensed kinetoplast disks, particularly in *L. tarentolae*.

The MitoCarta inventories of *T. brucei* (1,666 proteins) and *L. tarentolae* (1,594 proteins), generated by integrating our protein-correlation–profiling datasets with prior genome-wide fluorescent tagging studies^11,13^ and curated literature sources, document a substantial expansion of the kinetoplastid mitoproteome. In *T. brucei*, this refinement removed multiple false positives and added 124 previously unrecognized proteins. In *Leishmania*, the increase from the 388 proteins reported by Tasbihi et al. (2019)^23^ to 1,594 represents a major advance in coverage and accuracy. The comparable sizes of the two inventories reflect their shared mitochondrial architecture and metabolic complexity. These numbers substantially exceed those reported for human (1,136) and mouse (1,140),^76^ Arabidopsis (1,609),^77^ Acanthamoeba (1,122),^78^ and *S. cerevisiae* (901),^79^ placing kinetoplastid mitoproteomes among the most elaborate known. Much of this breadth derives from innovation within the Discoba, as 46% of the 1,503 mitochondrial orthogroups lack detectable homologs outside this clade.

Our MS analysis detected nine kDNA-encoded proteins in each organism. These highly hydrophobic proteins are translated from mRNAs spanning the full range of editing types, including never-edited, moderately edited, and pan-edited transcripts.^80^ Our results show that the extensive sequence heterogeneity of pan-edited mRNAs,^80^ such as COX3 and ATP6, does not perceptibly impair the abundance of their protein products, suggesting a translational control mechanism that discriminates between partially and fully edited mRNAs.

Notably, the protein product of the mitochondrial cryptogene unassigned to a known complex or function, MURF2, is robustly expressed in LtPMF and in both developmental stages of *T. brucei*. MURF2 has also been identified in other kinetoplastids, where it co-fractionates with Complex I subassemblies,^54^ indicating a possible functional association with the NADH dehydrogenase complex.

The *T. brucei* and *L. tarentolae* MitoCartas reveal extensive repertoires of mitochondrial proteins that lack homologs outside Kinetoplastea, conserved sequence or structural motifs, and functional annotation. This “dark matter” defines a rich frontier for discovery that may deepen our understanding of organellar evolution, specialization, and diversification across eukaryotes. Just as the mammalian MitoCarta^81^ transformed research in metazoans, these resources illuminate the remarkable complexity of the kinetoplastid mitochondrion and provide a foundation for future studies in parasite cell and structural biology and therapeutic targeting.

## LIMITATIONS OF THE STUDY

The MitoViaPCP inventories are estimated to have 90–93% sensitivity and a ~7% false discovery rate (cFDR). Based on these metrics, we expect ~110 false-positive assignments (e.g., several very low-abundance VSG proteins detected by DIA-MS) and a similar number of true mitochondrial proteins that were not captured, most likely due to low expression. We selected a PCP threshold (0.6) that prioritizes sensitivity, but we note that had we maximized “area under the curve” from our benchmark analysis, we would have selected a more stringent PCP threshold (0.86), leading to 90% sensitivity with 5% false discoveries for TbMitoViaPCP, which might represent a better trade-off. A further limitation of our inventory stems from the *T. brucei* TREU927 reference genome (TriTrypDB release 68), which contains a problematic contig (“Tb927_11_bin_v5.1”) enriched in pseudogenes. Fifty-five MitoCarta entries map to this contig, and each has a near-duplicate copy elsewhere in the genome, suggesting that these represent annotation artifacts rather than accurate gene models. We note that although immunoblotting confirmed that ATOM40 remained intact during purification, proteinase K treatment may nevertheless have reduced mass-spectrometric coverage of cytosol-exposed domains of other outer membrane proteins. Finally, functional annotations were derived in part from manual curation of prior literature, which may propagate existing inaccuracies or omissions.

## RESOURCE AVAILABILITY

### Lead contact

Requests for information and resources should be directed to the lead contact, Inna Aphasizheva (innaaf@bu.edu).

### Materials availability

Unique/stable reagents generated in this study are available from the lead contact.

### Data and code availability

- The mass spectra and the protein sequence databases used for searches have been deposited in MassIVE (MSV00010185). Confocal fluorescent microscopy and electron microscopy data and uncropped blots have been deposited at Mendeley Data at: https://doi.org/10.17632/2m586kg4z7.1. The TbMitoCarta and LtMitoCarta resources are available from the Mito-TOL website (www.mitocarta.org).
- All original code has been deposited at https://github.com/michaelzhuchen/mito-evolution and is publicly available as of the date of publication.
- Any other information required to reanalyze the data reported in this paper is available from the lead contact upon request.

## STAR METHODS

### EXPERIMENTAL MODELS AND STUDY PARTICIPANT DETAILS

Experiments were conducted with monomorphic procyclic and bloodstream form of *T. brucei brucei* Lister 427,^85^ and the UC strain of *Leishmania tarentolae*.^34^ *T. brucei* procyclic forms (TbPCF) were cultured in SDM-79 medium supplemented with 10% fetal calf serum (FCS) at 27 °C and harvested at a density of ~3 × 10^7^ cells/ml. TbBSF was maintained in HMI-9 medium containing 15% FCS at 37 °C and collected at ~2.4 × 10^6^ cells/ml. LtPMF (UC strain) was grown in Brain Heart Infusion (BHI) broth supplemented with 10 μg/ml hemin at 27 °C.

### METHOD DETAILS

#### Genome data annotation

Nuclear protein annotations were from TriTrypDB^65^ for *T. brucei* TREU927 (TriTrypDB-68_ TbruceiTREU927_AnnotatedProteins.fasta) and for *L. tarentolae* (TriTrypDB-68_Ltarentolae ParrotTarII_AnnotatedProteins.fasta). kDNA protein sequences and translations of published mRNA sequences^86,87^ were added. For the 128 *T. brucei* genes with multiple protein isoforms, we analyzed only the longest isoform. Protein sequences from both species were annotated with protein domains (HMMER version 3.1 using the Pfam-A database version 35^88^).

#### Published datasets

Six *T. brucei* datasets^11,12,14,18,21,24^ were integrated via *T. brucei* gene identifiers. For ROC analysis, TrypTag^11^ C-terminal tagging dataset (TrypTag-C), including proteins categorized as mitochondrial, kinetoplast, or antipodal, was further subdivided into: submito_Strong, mito_Strong, mito_dual_Strong, mito_Faint, mito_dual_Faint, nonmito_Faint, nonmito_Strong, and NA based keywords: submito = kinetoplast/antipodal, mito = mitochondrion, mito_dual indicates both mito and nonmito, and whether the signal was classified as faint (see Table S3). Moloney et al.^12^ PCF and BSF locations were downloaded (Table S9, column tagm.map.localisation.prediction) and sub-compartments combined based on compartment name. Panigrahi et al. (2009)^14^ proteomics data from *T. brucei* were downloaded from their Tables 1, 2, S5 and S6, corresponding to 887 identifiers. Of 388 *L. major* Friedlin protein sequences reported by Tasbihi et al. (2019),^23^ 374 were mapped to *L. tarentolae* proteins via BlastP top hit (Expect < 1e^−3^). The accuracy of each published dataset was assessed via benchmark analysis.

#### Benchmark analysis

*T. brucei* genes were partitioned into three annotation categories: “Gold+” (n = 546 genes with strong evidence of mitochondrial localization based on manual literature review), “Possible mito” (n=1,980 genes with TriTrypDB-62 GO annotations that mapped to “mitochondria” in GOslim or genes predicted mitochondrial by DeepLoc2.1^42^ with score > 0.4), or “Other“ (n =7,152 all other genes). Literature review for Gold+ was performed on all publications linked to GO slim “mitochondria” compartment in TriTrypDB-62, in addition to review articles and primary literature searches, to identify mitochondrial localization based on: (i) epitope-tagging and microscopy showing co-localization with a mitochondrial marker, (ii) fractionation and Western blot with proper controls, (iii) presence in a mitochondrial protein complex based on crystal structure or cryoEM, (iv) *in vitro* import assay with proper controls, or (v) kDNA-encoded. In addition, 72 proteins from Aphasizheva et al.^6^ were included in the Gold+ category based on expert curation linking these factors to mitochondrial RNA processing in *T. brucei*. To avoid circularity, proteins linked to mitochondria only through mass spectrometry studies^14,15,18,19,24,89–92^ were excluded from “Possible mito”. Annotations were mapped from *T. brucei* to *L. tarentolae* via orthology (BlastP version 2.2.30+ best bidirectional hits with Expect < 1e^−3^,^93^ yielding: 442 gold+ (BBH to *T. brucei* Gold+), 2,148 “Possible mito” (*L. tarentolae* DeepLoc mitochondrial score > 0.4 or BBH to *T. brucei* GO mito), or 5,880 other (all remaining genes).

Sensitivity, precision, and false discovery rate were calculated as described in Calvo et al. (2016)^94^ using true positives assigned from “Gold+” annotations and true negatives conservatively assigned from “Other” annotations: true positives (TP), true negatives (TN), false positives (FP), false negatives (FN), sensitivity SN = TP / (TP + FN), specificity SP = TN / (TN + FP), corrected false discovery rate cFDR = (1-SP)/(1-SP + SN x *O*_*prior*_), and Precision = 1 – FDR. *O*_*prior*_ = 0.17 prior probability of mitochondrial localization was estimated from the 1057/6058 (17%) proteins showing mitochondrial localization based on C-terminal tagging in the TrypTag study.^95^

#### Molecular cloning and transfections

Genomic modification of *T. brucei* was performed by in situ allele tagging through homologous recombination using PCR-generated templates encoding either eGFP–TS or a CTS (10His-Protein C-TwinStrep) tag.^38^ These constructs were used for mitochondrial immunoprecipitation or immunofluorescence microscopy. PCR products were transfected into Lister 427 PCF cells using the Human T-cell program of a 4D Nucleofector (Lonza) with P3 Primary Cell solution. DNA oligonucleotides are listed in Table S11.

#### Mitochondrial isolation

Mitochondria were isolated using either a density-gradient workflow (PK+DG) or an immunoprecipitation-assisted workflow (IP+PK+DG). The PK+DG protocol was applied to unmodified TbPCF and LtPMF cells, whereas the IP+PK+DG protocol was used for lines expressing epitope-tagged POMP10. In *T. brucei*, we introduced POMP10–eGFP–TS by in situ tagging; in L. tarentolae, we expressed POMP10–eGFP–TS from an episomal plasmid.^96^

OptiPrep is supplied as a 60% iso-osmotic iodixanol suspension in water. A 50% OptiPrep working solution was prepared by diluting the 60% stock with 0.6 M sorbitol, 120 mM Tris-HCl, and 12 mM EDTA (pH 7.4) supplemented with PMSF (freshly added from a 100 mM ethanol stock to 0.25 mM final) and an EDTA-free protease inhibitor cocktail (cOmplete Mini, Roche). Lower-percentage OptiPrep solutions (5–20% for *T. brucei* and 10–30% for *L. tarentolae*) were prepared from this working solution using 1× SoTE buffer (0.6 M sorbitol, 20 mM Tris-HCl, 2 mM EDTA, pH 7.4). Percentages refer to final iodixanol concentrations prepared from OptiPrep stock solutions. Continuous gradients were generated in open-top ultracentrifuge tubes (Seton Scientific, 14 × 89 mm, 7030) using a Gradient Master (Biocomp Instruments; for 10–30% gradients: 2 min, 80° angle, 20 rpm; for 5–20% gradients: 2:05 min, 81.5° angle, 12 rpm).

Cells were collected in logarithmic growth phase (~3.2 × 10^10^ TbPCF, ~1.6 × 10^10^ TbBSF, and ~1.2 × 10^11^ LtPMF total cells), washed with PBS, and resuspended in 50 mL of ice-cold SoTE supplemented with PMSF and protease inhibitors. Lysates were generated by nitrogen cavitation (85 bar for TbPCF and TbBSF, 90 bar for LtPMF) for 30 min at 4 °C. DNase I (40 U/ml) and MgCl_2_ (6 mM) were added and incubated on ice for 20 min with gentle swirling, followed by quenching with EDTA (6 mM). The membranous fraction was collected by centrifugation at 15,000×g for 15 min at 4 °C and resuspended in 50 mL SoTE. Unbroken cells were removed by one low-speed spin (560×g, 20 min, 4 °C) for *T. brucei* or two spins for *L. tarentolae*. Crude mitochondria were pelleted at 15,000×g for 15 min at 4 °C, resuspended in 10 mL of inhibitor-free SoTE, and split into PK(–) and PK(+) samples. PK(+) samples were treated with proteinase K (1 μg per 2.5 × 10^9^ TbPCF starting cells, 1 μg per 1.25 × 10^9^ TbBSF starting cells, and 2 μg per 2.5 × 10^9^ LtPMF starting cells) on ice for 10 min, quenched with PMSF at 1 mM, pelleted again, and washed twice in inhibitor-containing SoTE. Mitochondrial suspension (~0.3 ml) was applied on the top of OptiPrep gradient and centrifuged at 96,600×g for 30 min at 4 °C in SW41Ti rotor (Beckman) with slow brake. The upper mitochondrial band was collected by side puncture with an 18G needle, diluted fivefold in SoTE, and pelleted at 15,000×g for 15 min. Pellets were then frozen in liquid nitrogen, and stored at −80 °C.

#### Mitochondrial immunoprecipitation

Transgenic *T. brucei* and *L. tarentolae* expressing POMP10–eGFP–TS were harvested (~5.6 × 10^10^ TbPCF and ~1.2 × 10^11^ LtPMF cells), washed with PBS, and lysed by nitrogen cavitation (85 bar for *T. brucei*; 90 bar for *L. tarentolae*). DNase I/MgCl_2_ treatment, membranous fraction collection, low-speed clearing, and crude mitochondrial fraction collection were performed as described above. Crude mitochondria were incubated with 300 μl anti-GFP μMACS MicroBeads (Miltenyi Biotec) in 10 ml of SoTE for 1 h at 4 °C, and applied to paired LS MACS columns with 30 μm pre-filters on a QuadroMACS stand. After three washes in SoTE, bound material was eluted, pelleted at 15,000×g for 15 min, resuspended in 5 mL SoTE, and subjected to proteinase-K shaving (6 μg, 10 min on ice; quenched with PMSF). Samples were layered onto OptiPrep gradients and processed as above. Purified mitochondria were stored at −80 °C.

#### Protein quantitation and immunoblotting

Protein concentrations of whole-cell lysates and mitochondrial fractions were measured using the BCA Protein Assay Kit. Equal protein amounts were separated by SDS–PAGE, transferred to nitrocellulose membranes, and probed with antibodies listed in the Key Resources Table. Custom TbATOM40 and LtATOM40 antibodies target a conserved β-loop region (CTP2) present in Tom40 homologs from *Leishmania*, *Trypanosoma*, *Acanthamoeba*, and *Arabidopsis*. Additional antibodies included anti-biotin, anti-KRET1, anti-RESC1/2, anti-β-tubulin, and anti-GFP. Membranes were stripped and reprobed, as needed.

#### Confocal fluorescence microscopy

Proteins were selected for immunofluorescence validation based on comparative mitochondrial localization predictions. Four categories were analyzed. Category I included proteins assigned as mitochondrial in both MitoViaPCP inventories but annotated as non-mitochondrial in the TrypTag-C dataset; these were tested in *T. brucei*. Category II included proteins annotated as mitochondrial in TrypTag-C but not supported by either MitoViaPCP inventory; these were also tested in *T. brucei*. Category III included LtMitoViaPCP proteins lacking detectable *T. brucei* orthologs and was tested in *L. tarentolae*. Category IV included proteins identified as mitochondrial in only one MitoViaPCP inventory and annotated as mitochondrial or non-mitochondrial in TrypTag-C; these were tested in *L. tarentolae*. C-terminal CTS tagging was used to minimize interference with mitochondrial targeting.

CTS- or eGFP–TS-tagged proteins were detected using tag-specific antibody protocols. CTS-tagged proteins were labeled with StrepMAB-Immo as the primary antibody and Alexa Fluor 647–conjugated goat anti-mouse IgG (Invitrogen) as the secondary antibody. eGFP–TS-tagged proteins were detected using the Strep-TactinXT DY-488 conjugate. KRET1 (Tb927.7.3950) was analyzed independently of tagging using a monoclonal antibody,^97^ as previous work showed that C-terminal tagging did not yield reliable mitochondrial localization. Mitochondria and nuclear/kinetoplast DNA were labeled with MitoTracker Red CMXRos and Hoechst 33342, respectively. Images were acquired on a Nikon Ni2 AX inverted confocal microscope equipped with Digital Sight 10 and Digital Sight 50M cameras using a CFI Plan Apochromat VC 60×C oil-immersion objective (NA 1.42). Z-stacks spanning each cell were acquired and deconvolved using NIS-Elements (v. AR 5.41.02).

#### Transmission electron microscopy

Mitochondrial and whole-cell pellets were fixed in 2.5% glutaraldehyde and 2.5% formaldehyde in 0.1 M sodium cacodylate buffer (pH 7.4) for at least 2 h at room temperature. Samples were washed in cacodylate buffer, post-fixed in 1% osmium tetroxide with 1.5% potassium ferrocyanide for 1 h at room temperature, washed, and incubated in 1% uranyl acetate prepared in 50 mM maleate buffer (pH 5.15) for 1 h. Samples were dehydrated through graded ethanol, incubated in propylene oxide for 1 h, infiltrated overnight in a 1:1 mixture of propylene oxide and TAAB Epon resin, embedded in fresh resin, and polymerized at 60 °C for 48 h. Ultrathin sections (80 nm) were cut on a Reichert Ultracut S ultramicrotome, mounted on copper grids, stained with lead citrate, and imaged on a Tecnai Spirit BioTwin transmission electron microscope equipped with an AMT NanoSprint43-MkII camera.

#### Preparation of samples for mass spectrometry

Samples were incubated on ice for 15 min in SDS lysis buffer (5% SDS, 50 mM TEAB pH 8.5, 2 mM MgCl_2_, 2 μg/mL Aprotinin, 10 μg/mL Leupeptin, 1 mM PMSF). After lysis, DNA in each sample was degraded with 3 μL of 250 U/μL benzonase. Lysates were then clarified by centrifugation at 20,000 g, 10 °C for 10 min, and supernatants were transferred into a separate tube. Protein concentration was quantified using a BCA protein assay.

Disulfide bonds in the supernatant were reduced in 5 mM DTT at 25 °C with shaking at 1,000 rpm for 1 hr, followed by alkylation of cysteine residues in 10 mM IAA at 25 °C with shaking at 1,000 rpm for 45 min in the dark. Samples were eventually acidified by adding 12% phosphoric acid at a 1:10 (*v/v*) ratio (acid:lysate).

Acidified lysates were precipitated with ice-cold S-Trap buffer (90% methanol, 100 mM TEAB) at a 6:1 (*v/v*) ratio (buffer:lysate). The precipitated protein mixture was loaded onto S-Trap columns (Protifi), with column size selected based on total protein input, and centrifuged at 3,000 g for 1 min. Proteins bound to the column were washed four times with S-Trap buffer by centrifugation at 3,000 g for 1 min, and were then digested overnight at 25 °C with digestion buffer (50mM TEAB with trypsin and LysC, each at a 1:50 ratio to proteins, *w/w*). Specifically, the digestion buffer was passed through the column once by centrifugation at 2,500 g for 1 min and added back onto the column for the overnight digestion. Hereafter, the digested peptides were eluted sequentially with 50mM TEAB, 0.1% FA, and twice of 50% ACN/0.1% FA by centrifugation at 3,000 g for 1 min. Eluates were snap-frozen and vacuum-centrifuged until dry.

Dried peptides were reconstituted in 1% FA and desalted using 40 mg Sep-Pak tC18 96-well plates (Waters, SKU:186002320). C18 wells were conditioned sequentially with 100% ACN, 50% ACN/0.1% FA, and four washes of 0.1% TFA. Peptides were loaded onto the wells and washed three times with 0.1% TFA, followed by one wash with 1% FA. Eventually, the desalted peptides were eluted twice with 50% ACN/0.1% FA. Eluates were snap-frozen and vacuum-centrifuged until dry. The dried, desalted peptides were reconstituted in 30% ACN/0.1% FA, and peptide concentrations were quantified using a BCA assay before aliquoting for LC-MS/MS DIA analysis.

#### LC–MS/MS analysis

All peptide samples were reconstituted in 3% ACN/0.1% FA for LC-MS/MS analysis. Peptides were analyzed on an Orbitrap Astral mass spectrometer (Thermo Fisher Scientific) coupled to a Vanquish Neo UHPLC system (Thermo Fisher Scientific). 500 ng of peptides from each sample were injected onto a PepSep C18 column (Bruker Daltonics, 25cm length x 75 μm inner diameter, 1.5 μm particle size) with a flow rate of 300nL/min. Samples were run on a linear 47.5 minute gradient from 4.6% solvent B (99.9% ACN, 0.1% FA) to 24% solvent B before ramping up to 48% solvent B over 4 minutes. To wash the column, solvent B was increased to 72% for 7.5 minutes. MS1 spectra were measured with a resolution of 240,000 at 200 m/z and a mass range from 300 to 1800 m/z. The maximum injection time for MS1 was set at 5 ms and with an AGC target of 2E6. MS1 spectra were acquired with a timed loop control of 0.6 s. MS2 scans covered a mass range of 380–980 m/z with an isolation window of 4 Th. The maximum injection time was set to 5 ms for DIA scans, with an AGC target of 5E5. Precursors were fragmented with higher-energy collisional dissociation (HCD), with a normalized collision energy (NCE) set to 27%.

#### Mass spectrometry data processing

MS spectra were searched using Spectronaut 19. Data were searched against annotated proteins databases for *Trypanosoma brucei brucei* TREU927 and *Leishmania tarentolae* Parrot-TarII reference strains (https://tritrypdb.org/tritrypdb, release 68) in directDIA mode using quantification settings with a precursor Q-value cutoff of 0.01, a precursor PEP cutoff of 0.2, protein Q-value cutoff (experiment) of 0.01, a protein Q-value cutoff (run) of 0.05, and a protein PEP cutoff of 0.75. The MS/MS search included the fixed modification Carbamidomethyl (C) and variable modifications: Oxidation (M), Acetyl (Protein N-term), Deamidation (N), and FormylMet (Protein N-term), with a maximum variable modification count of 5. Best N fragments were set as “3 to 15”. Results reported total MS2 intensities for each protein group. Column sum normalization was applied across samples (normalized_intensity_*g,s*_ = scalingFactor * intensity_*g,s*_ /columnSums, for protein group *g* and sample *s*, where columnSums is the sum of intensities of sample *s*, and scalingFactor = mean(columnSums for all *s*). Next, protein groups were expanded such that normalized_intensity_*p,s*_= normalized_intensity_g,s_ for all proteins *p* in protein group *g*. Normalized intensities were log10-transformed and then MinProbAll imputation was performed (missing values were replaced with values drawn from a Gaussian with mean set to the 1st percentile of values across all samples, and sigma set to the median of standard deviations of each protein across all samples). Z-scores were computed for each protein across all samples.

#### Protein correlation profiling (PCP) analysis and MitoCarta compendium

PCP scores were calculated using the Pearson correlation between the Z-score profile for each protein versus the mean of three mitochondrial markers (Tom40/Tb927.9.9660/LtaP35.4850; Hspd1/Tb927.10.6510/LtaP36.2020; IscU/Tb927.9.11720/LtaP35.3670). Genes with PCP score ≥ 0.6 were labeled “MitoViaPCP.” MitoCarta was defined as a union of MitoViaPCP (Tb PCP threshold 0.6, Lt PCP threshold 0.6), gold+ literature, and TrypTag C^11^ localization to mitochondria, excluding proteins with Tb PCP scores < 0 (and excluding five genes, which manual review of TrypTag images showed to be faint or inconsistent: Tb927.1.270, Tb927.4.1200, Tb927.4.5360, Tb927.5.2020, Tb927.6.2310).

#### Pathway annotation

Functional annotation of *T. brucei* mitochondrial proteins was performed by manual literature review. Pathways were organized using a three-level hierarchy adapted from MitoCarta3.0,^76^ beginning with seven top-level modules (Small_molecule_transport, Signaling, Protein_import_ sorting_and_homeostasis, OXPHOS, Mitochondrial_dynamics_and_surveillance, Mitochondrial_ central_dogma, and Metabolism). These modules were expanded into 21 level 2 categories and 56 level 3 categories. PubMed and Google Scholar searches were performed with pathway and enzyme-specific keywords, and review articles were consulted to establish pathway boundaries. Proteins were included only when at least one primary publication provided experimental evidence consistent with the proposed function (Table S3).

Mitochondrial protein complexes were curated using complex-specific literature searches in PubMed and Google Scholar. Review articles were used to identify candidate complexes and subunits, and proteins were classified as bona fide components only when supported by at least one of the following criteria: identification by mass spectrometry after immunoprecipitation of a validated bait protein, visualization in a cryo-EM structure, or consistent detection across multiple independent studies. Curated complex annotations are reported in Table S3 and were used for all analyses.

#### Homology across species

Homology between *T. brucei* and *L. tarentolae* proteins was determined based on BLASTP^93^ best bidirectional hit (E<1) and orthogroups (OGs). Homology to model organisms was assigned using OGs and via BLASTP^93^ version 2.2.30+ to *Homo sapiens* (RefSeq release 63, using longest transcript per Entrez gene), *S. cerevisiae* S288C (*Saccharomyces* Genome Database^98^) and *A. thaliana* (Araport11 from TAIR).^99^ Homology of *T. brucei* and *L. tarentolae* proteins to 203 eukaryotic species, 29 bacterial clades (grouped at superfamily-level), and 5 archaeal clades (grouped at kingdom-level) was determined based on OGs that were inferred by OrthoFinder^100^ (v2.5.5) and refined using a custom pipeline incorporating profile Hidden Markov Model and Alphafold structure alignments.^64^

#### Expression across species

In Figure 6A, MS intensity data from three whole-cell LtPMF were compared with those from TbPCF and TbBSF, without normalization between replicates or datasets. In Figure 6B, MS intensity data from replicate 1 of whole cell lysate from each species was limited to TbMitoCarta and LtMitoCarta orthologs (BBH) and then column sum normalized and log-10 transformed, and then GSEA was performed using genes pre-ranked by significance score (−log10(p-value from t-test across species, with sign flipped for log2fc<0) using manually annotated pathways.

#### Evolutionary analyses

The putative ancestry of each protein was assigned based on the presence/absence of its orthogroup across species. First, proteins with homologs in at least two prokaryotic clades were labeled as “Prokaryotic.” Second, proteins with homologs in at least two eukaryotic superfamilies (requiring homologs in at least two species within each superfamily) and in orthogroups containing > 90% eukaryotic species were labeled as “Eukaryotic.” Third, proteins with no identified homologs beyond Discoba were labeled as “Discoba-specific.” The latter represent a mixture of truly lineage-specific innovations and proteins too short or fast-evolving for accurate homology detection. The subset powered to detect distant homologs based on sequence alignment of Discoba homologs was defined using abSENSE-HMM.^64^

#### Species tree

The species tree used for visualization was inferred from a concatenated alignment of universal ribosomal proteins across eukaryotes, bacteria, and archaea using a manually-refined NCBI taxonomy as a guide tree.^64^

### QUANTIFICATION AND STATISTICAL ANALYSIS

We performed statistical analyses in Excel and R. Details of the benchmark analysis of published and mitochondrial proteomics datasets generated in this study are provided in the Methods and include how benchmark datasets were created, size-corrected, and used to determine the sensitivity and precision of the MitoViaPCP list and published inventories. Two-sided Student’s t-tests (shown in volcano plots and barplots in the figures) were performed using the function t.test in Excel and R on input data that met assumptions of normal distribution based on manual inspection of underlying histograms.

## Supplementary Material

TableS1 **Table S1.** Published and newly generated *T. brucei* mitoproteome datasets, benchmark labels, and benchmark statistics, related to Figure 1

TableS3 **Table S3.** *T. brucei* gene-based summary of mitochondrial Protein Correlation Profiling (PCP) results for all proteins (Tab A), the MitoViaPCP list (Tab B), TbMitoCarta (Tab C) and predicted TbDualMito (Tab D). All data correspond to the procyclic form, related to Figure 1

TableS4 **Table S4.** Benchmark statistics corresponding to receiver-operator curves (ROC), related to Figures 1, 2, 3, S1 and S2

TableS2 **Table S2.** DIA-MS Intensity and iBAQ values for Protein Groups (tab A) and Peptides (tab B) corresponding to the mitochondrial serial purification and MitoIP experiment for TbPCF, related to Figure 1

TableS5 **Table S5.** DIA-MS Intensity and iBAQ values for mitochondria purified from TbBSF (tabs A, B) and comparison of whole cell lysate abundance in procyclic versus bloodstream forms (tabs C, D), as well as a gene-based summary across datasets (tab E), related to Figure 2

TableS6 **Table S6.** DIA-MS Intensity and iBAQ values for Protein Groups (tab A) and Peptides (tab B) corresponding to the mitochondrial purification from LtPMF, related to Figure 3

TableS8 **Table S8.** Protein (tab A) and peptide (tab B) data for kinetoplast DNA-encoded proteins across all *T. brucei* and *L. tarentolae* experiments, related to Figure 5

TableS9 **Table S9.** DIA-MS abundance of MitoCarta orthologs in whole cell lysate in *T. brucei* and *L. tarentolae* insect stages, related to Figure 6

TableS10 **Table S10.** Orthologous group (OG) assignments and comparative mitochondrial annotation analyses across *T. brucei*, *L. tarentolae*, human, yeast, and Arabidopsis, including all OGs (tab A), MitoCarta-associated OGs (tab B), kinetoplastid-enriched mitochondrial PFAM domains (tab C), and OG analysis of GDM and GMER pathways (tab D), related to Figure 7

TableS11 **Table S11.** DNA oligonucleotides used in this study

TableS7 **Table S7.** *L. tarentolae* gene-based summary of mitochondrial Protein Correlation Profiling (PCP) results for all proteins (tab A), the MitoViaPCP list (tab B), and LtMitoCarta (tab C), related to Figure 3

SuppMaterials

Supplemental Data 1

**Document S1.** Figures S1–S4

## Figures and Tables

**Figure 1.**
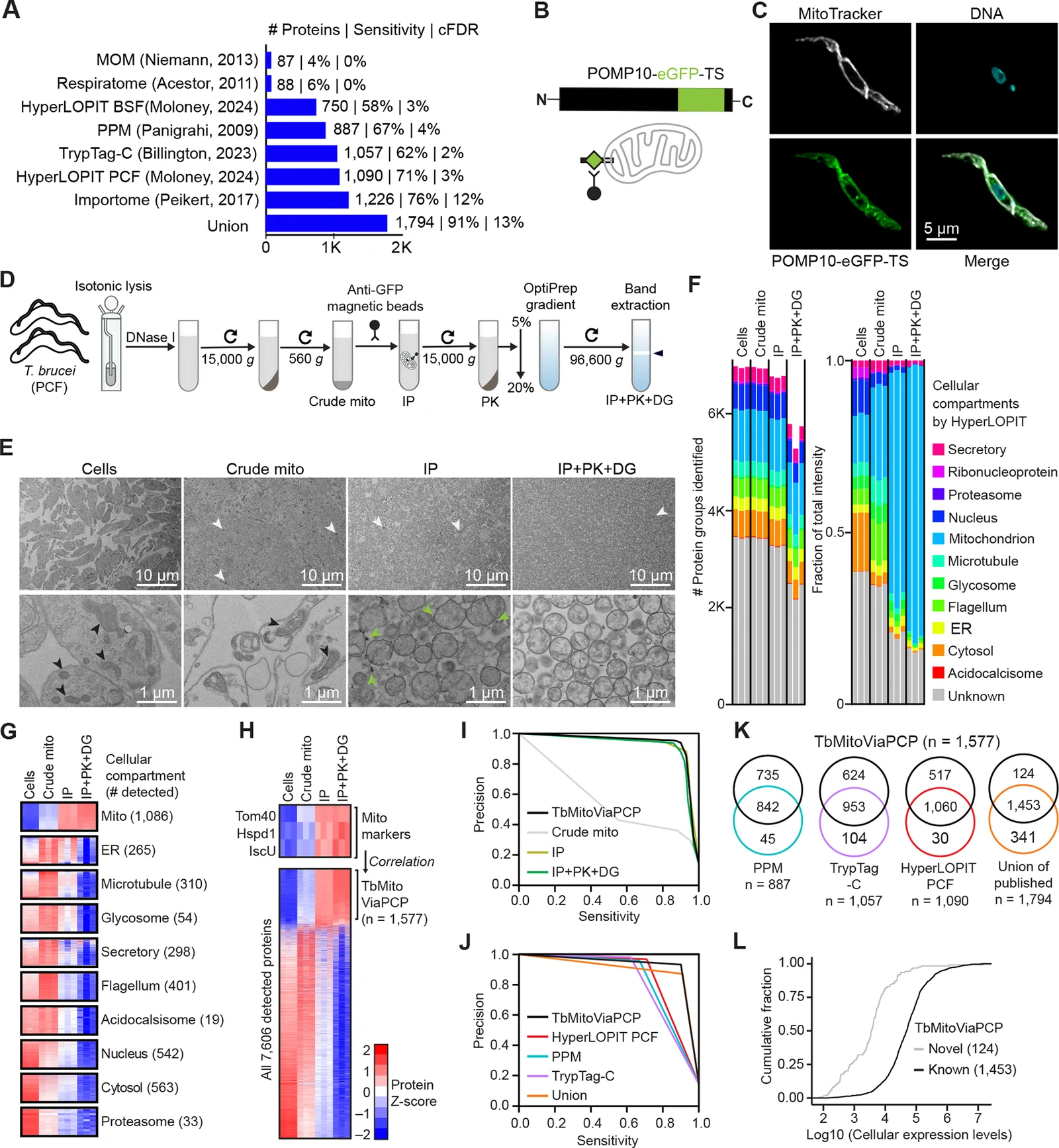
Protein correlation profiling yields a comprehensive and accurate mitochondrial proteome of the insect form of *T. brucei* (A) Summary of *T. brucei* mitoproteome datasets from published studies, showing the number of proteins, sensitivity, and corrected false discovery rate (cFDR) for each dataset. (B) Schematic of the MOM protein POMP10 C-terminally tagged with eGFP–Twin-Strep (TS). (C) Confocal fluorescence microscopy of POMP10–eGFP–TS in TbPCF. (D) Workflow for mitochondrial purification from TbPCF. Crude mito, membranous fraction recovered from the cell lysate by centrifugation; IP, material eluted from immunoaffinity magnetic beads; IP + PK + DG, immunoprecipitation followed by proteinase K treatment (PK) and density-gradient centrifugation (DG). Recovered band is indicated. (E) Transmission electron micrographs of sequential purification steps. Green arrowheads indicate anti-GFP magnetic nanobeads; white arrowheads indicate kinetoplast DNA disks; black arrowheads indicate mitochondria. (F) Number of protein groups (left) and fraction of total intensity (right) across purification steps. Protein groups are colored by subcellular compartment as annotated by HyperLOPIT.^12^ (G) Distribution of proteins across purification steps. Relative abundances of proteins (rows) quantified by DIA-MS are aligned with subcellular compartments as annotated by HyperLOPIT. (H) Relative abundances are shown for three marker proteins used across the Mito-TOL project (top) and all detected proteins sorted by PCP score (Pearson correlation with the average profile of three mitochondrial marker proteins). Proteins with PCP ≥ 0.6 are designated TbMitoViaPCP. (I) Sensitivity-precision curves for fractions of increasing mitochondrial enrichment (log_2_(fraction/ whole cell, single replicates)) vs. TbMitoViaPCP. (J) Sensitivity–precision curves for published mitoproteomes for procyclic *T. brucei*^11,12,14^ vs. TbMitoViaPCP. (K) Venn diagrams showing overlap among TbMitoViaPCP, published mitoproteomes, and the union of published datasets.^11,12,14,18,21,24^ (L) Cumulative distribution of cellular protein expression (log_10_ iBAQ) for TbMitoViaPCP proteins partitioned into those that overlap the aggregated published mitoproteomes and identified proteins. For visualization, missing whole-cell abundance values were imputed for identified proteins not detected by whole-cell DIA-MS, enabling inclusion of the complete 124-protein set.

**Figure 2.**
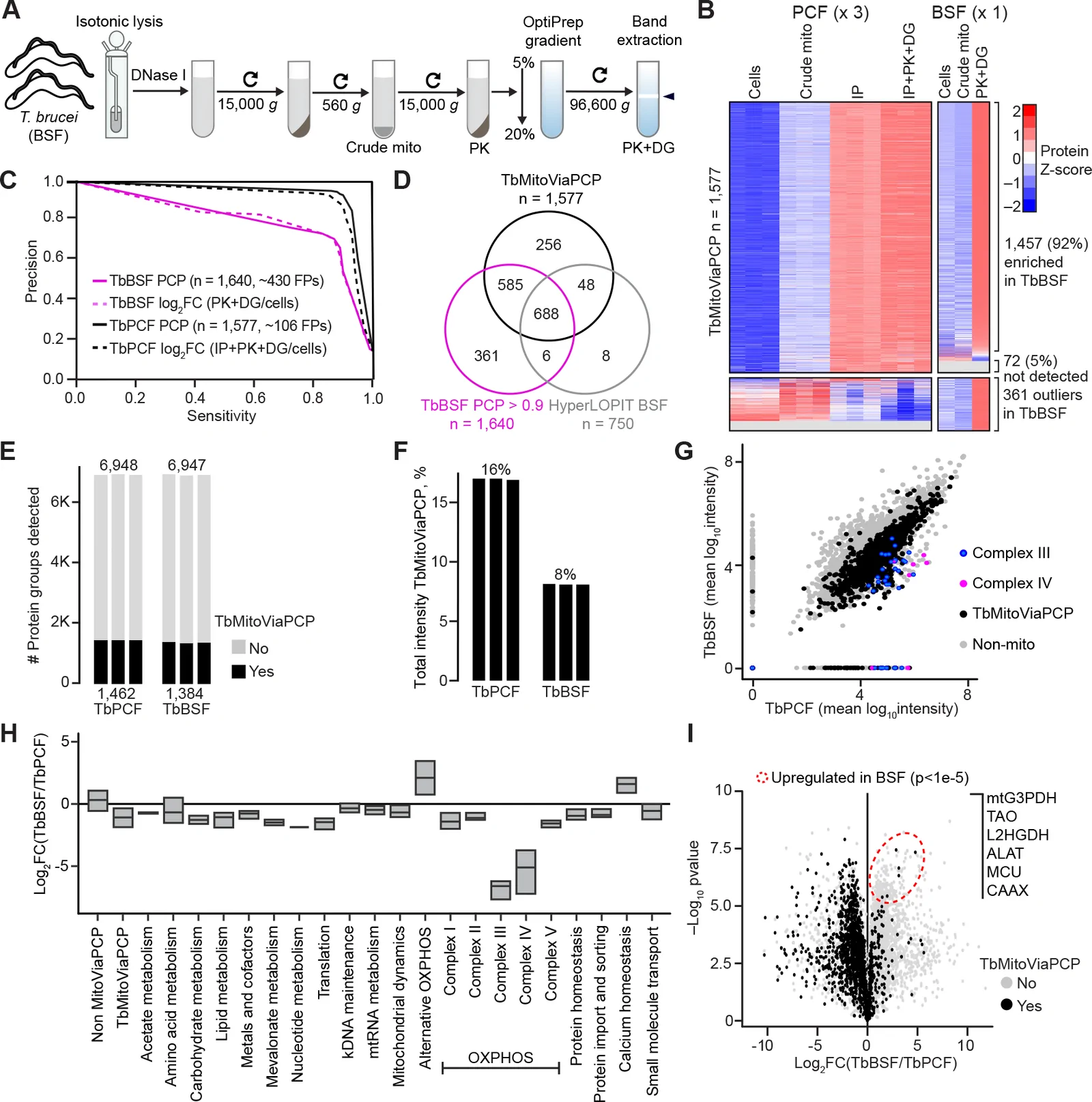
The mitoproteome is conserved across insect and bloodstream forms of *T. brucei* (A) Workflow for mitochondrial purification from TbBSF. (B) Relative protein abundances quantified by DIA-MS analysis of samples across TbPCF (left) and TbBSF (right) mitochondrial purification workflows. The top panels show all TbMitoViaPCP proteins ordered by decreasing TbBSF PCP score (correlation with the average profiles of marker proteins Tom40, Hspd1, and IscU). Bottom panels show 361 high-scoring proteins in TbBSF (PCP > 0.9) not present in TbMitoViaPCP, ordered by decreasing TbPCF PCP score. (C) Sensitivity–precision curves for TbBSF and TbPCF datasets, including PCP scores (solid lines) and log_2_(fraction/whole cell) values from single replicates (dashed lines). FP, false positive. (D) Venn diagram showing the overlap among TbBSF proteins with PCP score > 0.9, TbMitoViaPCP, and HyperLOPIT BSF datasets. (E) Number of proteins detected in TbPCF and TbBSF whole-cell lysates, and the mean number of TbMitoViaPCP proteins identified across three replicates. (F) Total intensity of TbMitoViaPCP proteins in whole-cell triplicates for TbBSF and TbPCF. (G) Mean protein abundances (log_10_ intensity across three replicates) measured by whole-cell DIA-MS are plotted for TbMitoViaPCP proteins in procyclic (TbPCF; X-axis) and bloodstream (TbBSF; Y-axis) forms. Each point represents one protein. (H) Boxplot showing the abundance distributions (25th–75th percentile) of individual proteins across 15 mitochondrial pathways, five OXPHOS complexes, and two alternative respiratory units (TAO and NDH2). (I) Volcano plot showing fold change (TbPCF/TbBSF) versus significance (–log_10_ *p*-value, *t*-test) for all detected proteins.

**Figure 3.**
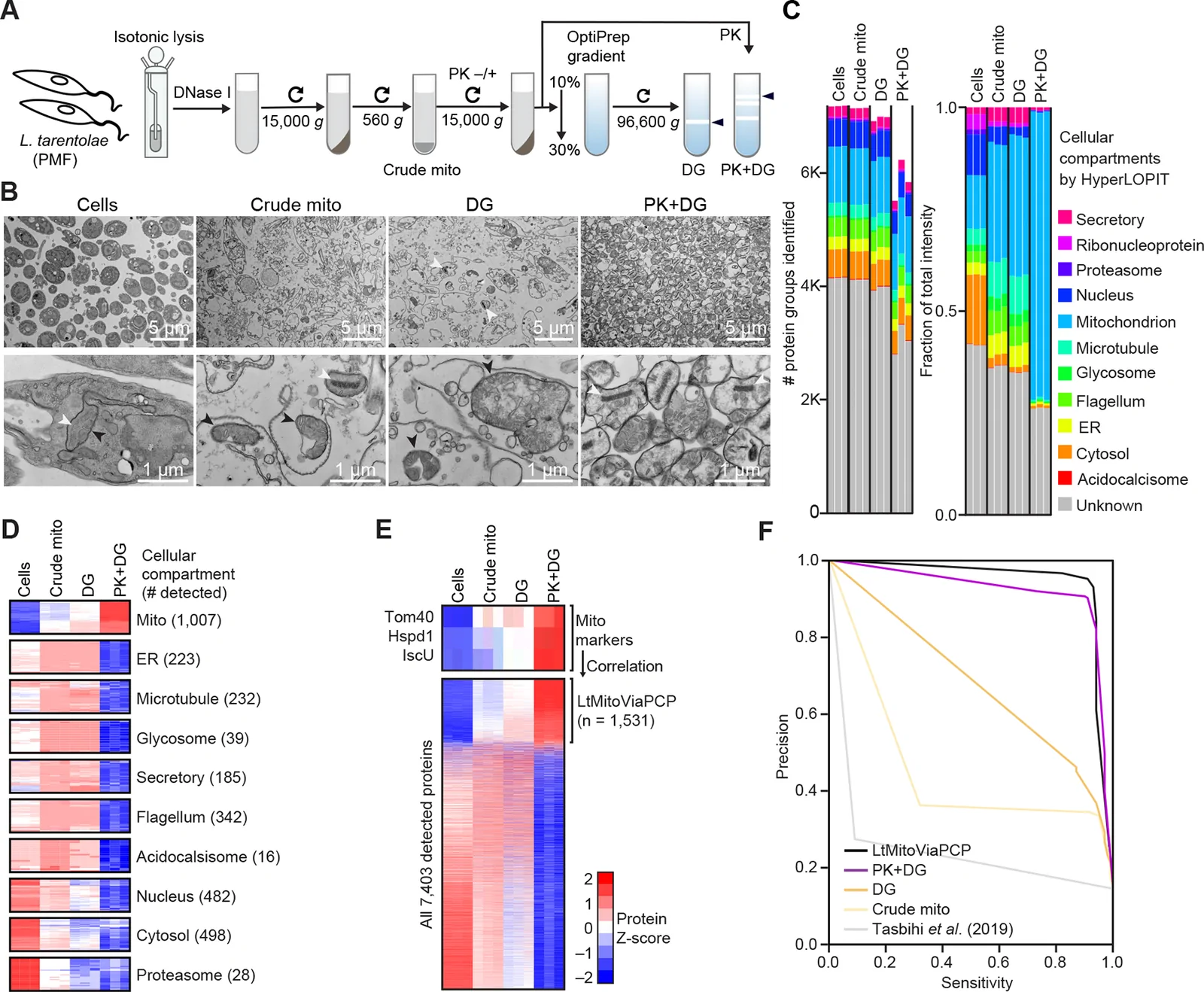
Serial enrichment yields highly purified *L. tarentolae* mitochondria (A) Workflow for mitochondrial purification from LtPMF. Crude mito, membranous fraction; DG and PK+DG, buoyant fractions recovered from gradient-separated mock-treated or proteinase K-treated membranous fractions, respectively. See also Figure S3C. (B) Transmission electron micrographs of sequential purification steps. White arrowheads indicate kinetoplast DNA disks and black arrowheads indicate mitochondria. (C) Number of protein groups identified and quantified by mass spectrometry in fractions of increasing mitochondrial purity (left) and their corresponding fractions of total intensity (right). Colors indicate the subcellular localization of *Leishmania* orthologs mapped to the TbPCF HyperLOPIT dataset. (D) Relative protein abundances across purification steps. Rows represent proteins grouped by HyperLOPIT-predicted subcellular localization in *T. brucei*, mapped to *Leishmania* orthologs. Numbers in parentheses indicate the number of *L. tarentolae* proteins per compartment. (E) Relative protein abundances sorted by PCP score (Pearson correlation with the average profile of three conserved mitochondrial marker proteins shown at top). Proteins with PCP score ≥ 0.6 were designated as the LtMitoViaPCP dataset. (F) Sensitivity–precision curves [log_2_(fraction/whole cell)] for LtMitoViaPCP, fractions from the mitochondrial enrichment workflow, and the *L. tropica* mitoproteome dataset from Tasbihi *et al.* (2019).^23^

**Figure 4.**
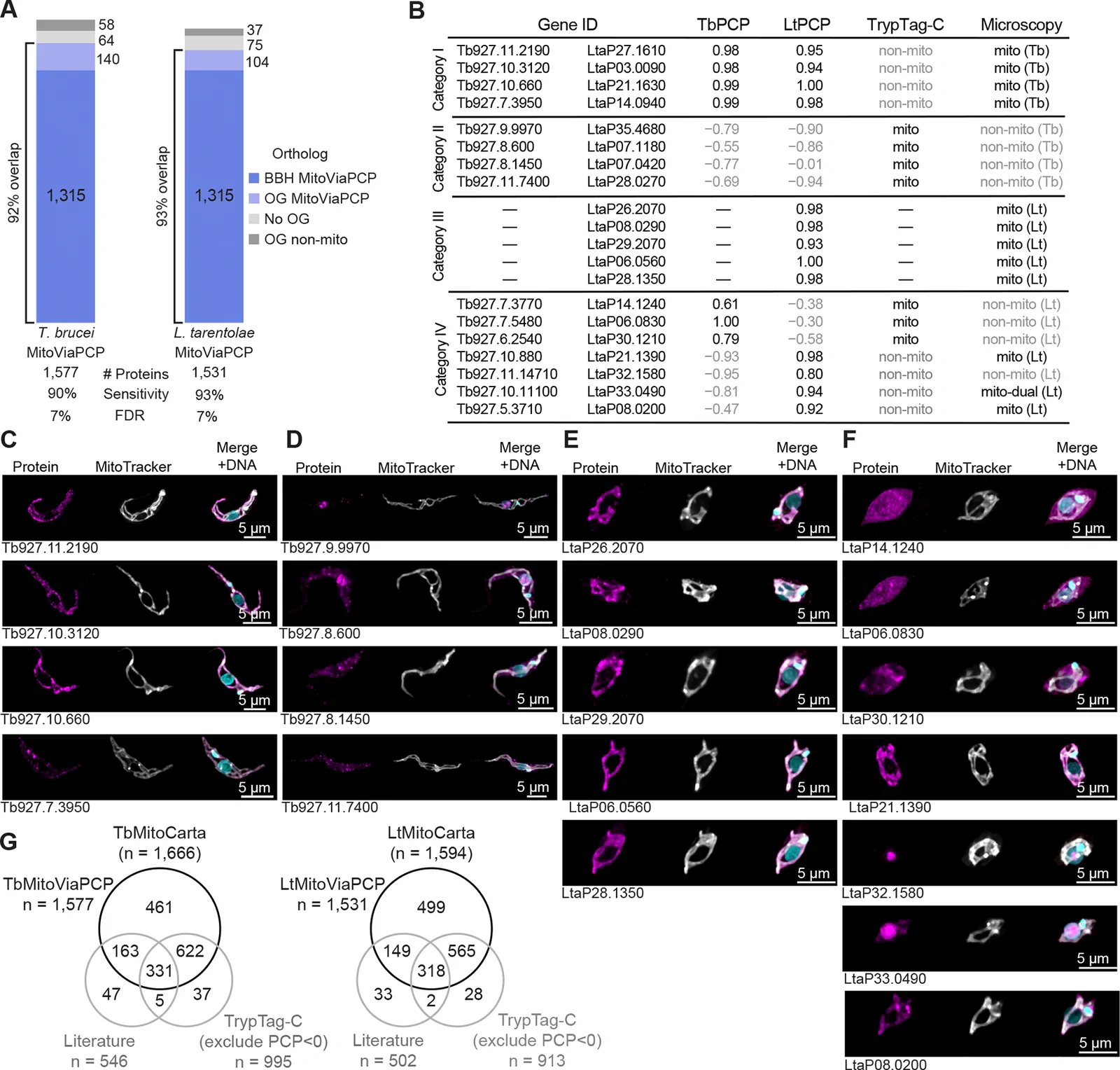
Overlap between mitochondrial proteomes of *T. brucei* and *L. tarentolae* (A) Overlap between mitochondrial inventories based on BLASTP best bidirectional hits (BBH) or orthogroup (OG) assignment. Sensitivity and false discovery rate (FDR) values are derived from benchmark analyses. (B) Summary of validation outcomes for MitoViaPCP datasets in *T. brucei* and *L. tarentolae*. Protein assignments from PCP and TrypTag-C, and microscopy (mitochondrial or non-mitochondrial) are tabulated. Categories (I)–(IV) correspond to panels (C)–(F). (C) Category I: proteins identified as mitochondrial in both MitoViaPCP datasets but annotated as non-mitochondrial in TrypTag-C; validated in TbPCF. (D) Category II: proteins identified as non-mitochondrial in both MitoViaPCP datasets but annotated as mitochondrial in TrypTag-C; validated in TbPCF. (E) Category III: proteins unique to the *L. tarentolae* MitoViaPCP dataset with no detectable *T. brucei* homologs; validated in LtPMF. (F) Category IV: proteins classified as mitochondrial by either TbMitoViaPCP or LtMitoViaPCP; validated in LtPMF. (G) Venn diagrams illustrate the construction of the MitoCarta inventories from MitoViaPCP, TrypTag-C, and curated literature datasets.

**Figure 5.**
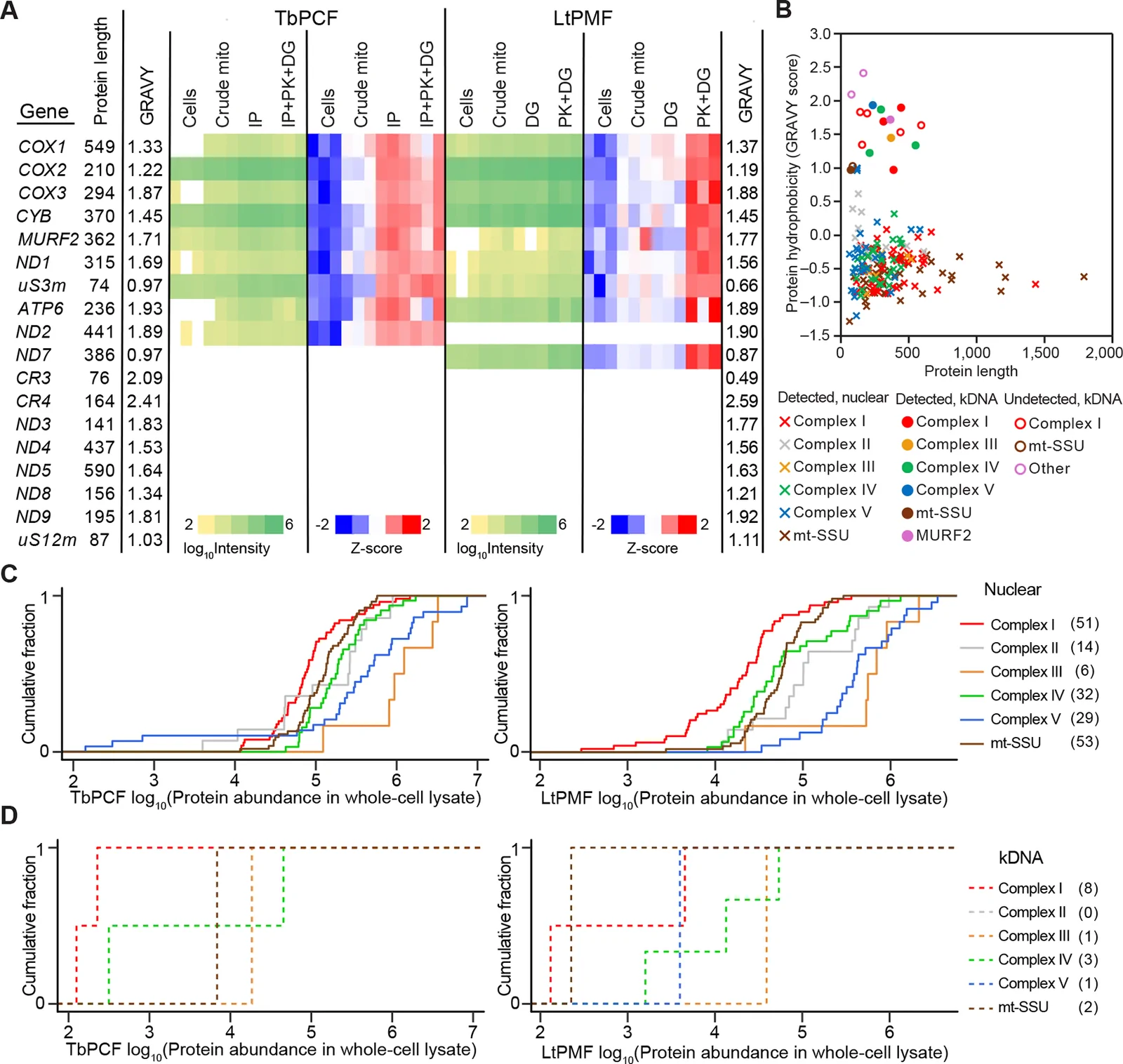
Kinetoplast DNA-encoded proteins in *T. brucei* and *L. tarentolae* (A) Heatmap of kDNA-encoded proteins identified across mitochondrial enrichment steps, including eight shared between species and two uniquely detected in either organism. Values represent normalized log_10_ intensities and *z*-scores. GRAVY (Grand Average of Hydropathy)^82^ scores are shown alongside. (B) Scatter plot of GRAVY score versus protein length for nuclear- and kDNA-encoded proteins, grouped according to their respective complexes. (C) Cumulative distribution plots show the abundance of nuclear-encoded complex subunits in whole-cell lysates. The number of annotated subunits in *T. brucei* is indicated in parentheses. (D) Cumulative distribution plots showing the abundance of kDNA-encoded complex subunits in whole-cell lysates. The number of annotated subunits in *T. brucei* is indicated in parentheses.

**Figure 6.**
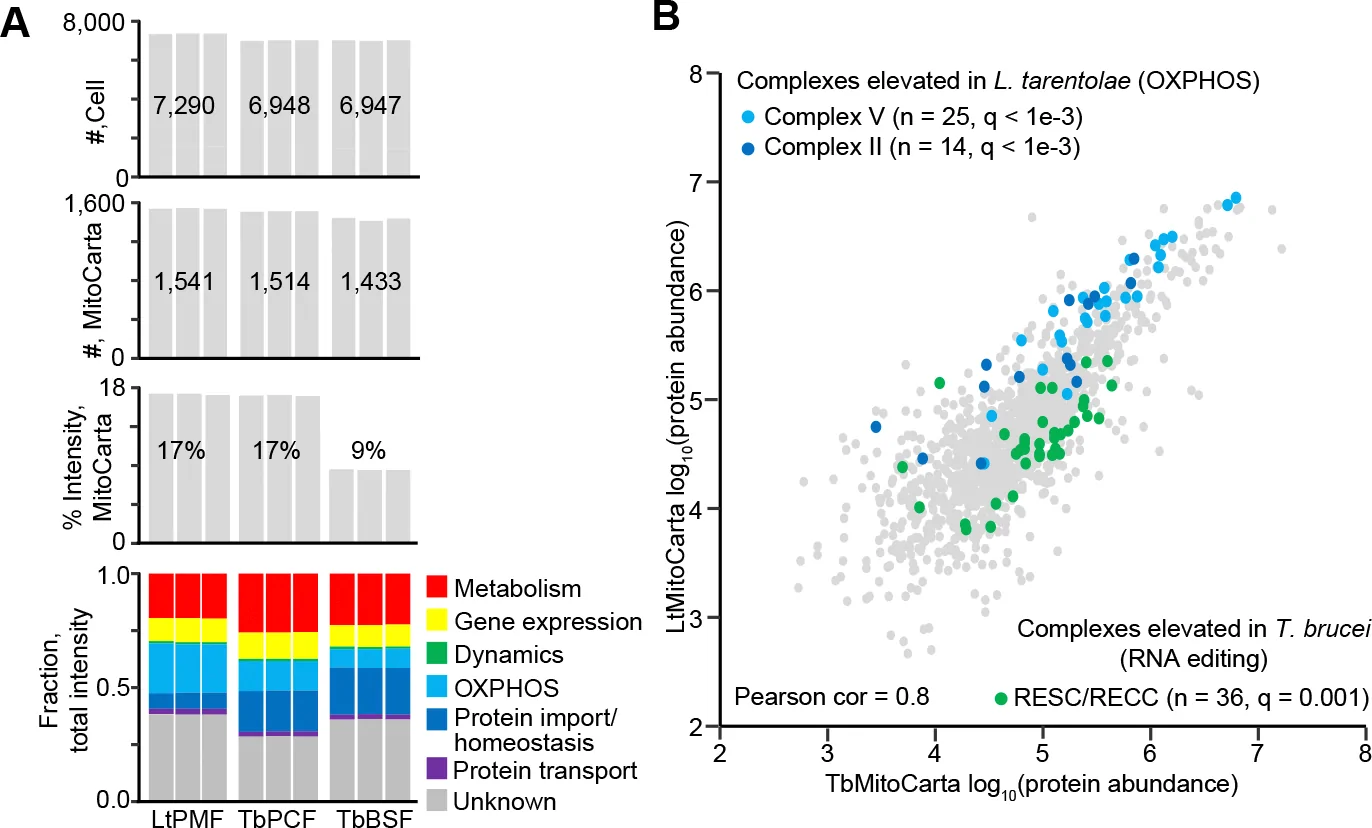
MitoCarta expression is similar across *L. tarentolae* and *T. brucei* insect stages (A) Bar plots show the total number of proteins detected, the number of MitoCarta proteins, the percentage of MS intensity derived from MitoCarta proteins, and the fraction of MitoCarta intensity partitioned by functional category in whole-cell lysate replicates. (B) Scatter plot shows abundance in cell lysate for 1,329 LtMitoCarta proteins and their orthologs in TbMitoCarta (mean of three replicates). RESC, RNA editing substrate binding complex.^39^ RECC, RNA editing catalytic complex.^83^ Protein abundance was normalized within this subset and then log_10_ transformed. Three gene sets for complexes that were significantly differentially expressed based on gene set enrichment analysis (GSEA)^57,58^ are highlighted in color.

**Figure 7.**
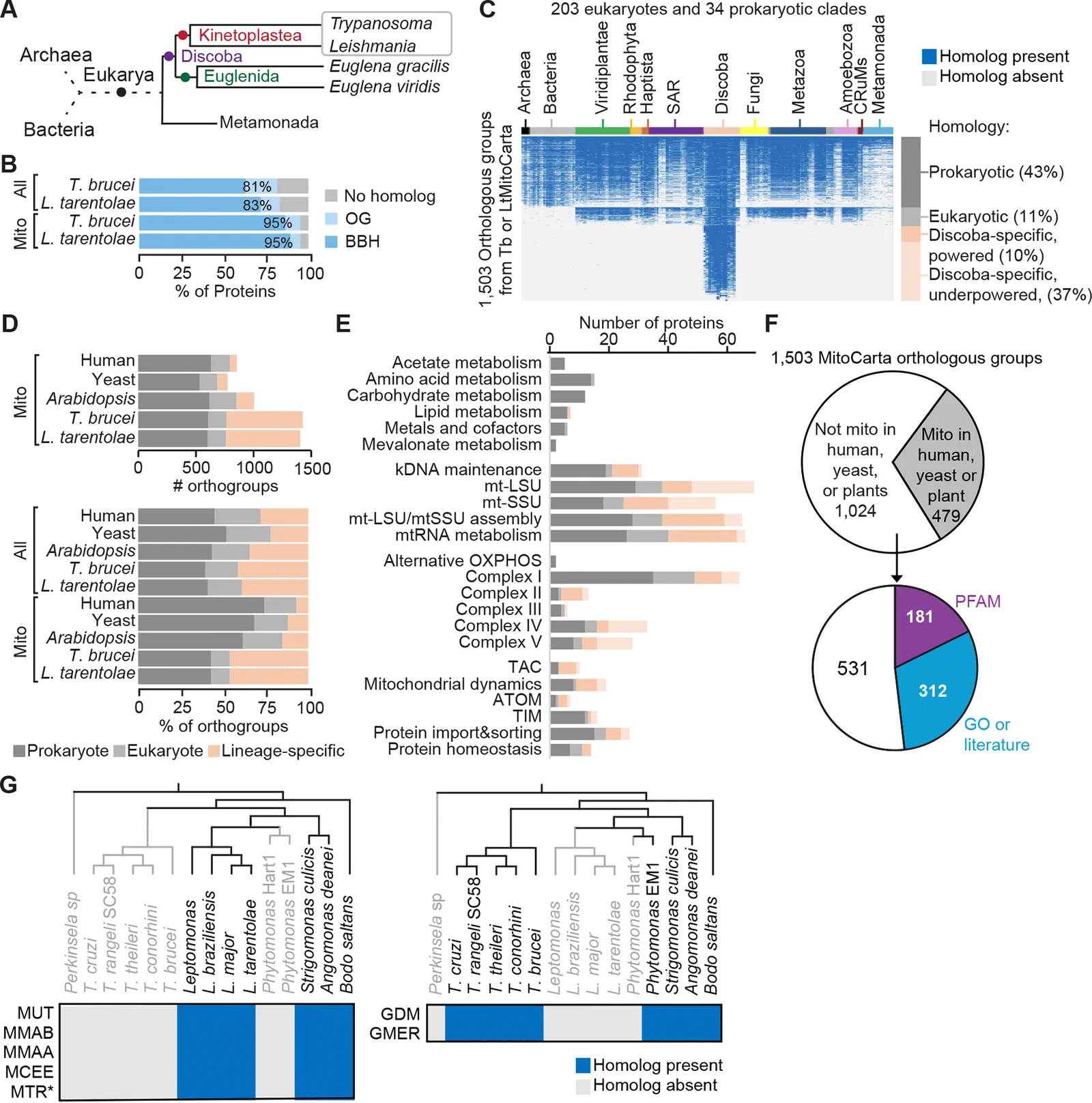
Evolutionary and pathway divergence of kinetoplastid mitochondrial proteomes (A) Phylogenetic placement of *Trypanosoma* and *Leishmania* within class Kinetoplastea and the clade Discoba, with the Metamonada outgroup. (B) Stacked bar plots illustrating homolog presence between *T. brucei* and *L. tarentolae*, defined by best-bidirectional BLASTP hits or orthologous-group (OG) membership. (C) Heatmap showing the presence (blue) or absence (gray) of all OGs containing MitoCarta proteins from *T. brucei* or *L. tarentolae* (rows) across eukaryotic and prokaryotic species (columns). Homology categories on the right denote OGs with homologs in prokaryotes, eukaryotes, or restricted to Discoba. Discoba-restricted OGs include lineage-specific innovations and proteins that are too short or fast-evolving for reliable homology detection (underpowered). (D) Stacked bar plots summarizing OG homology to prokaryotes and eukaryotes for mitoproteomes of selected species. (E) Stacked bar plots showing OG homology for literature-based mitochondrial pathway annotations in *T. brucei*, with colors corresponding to homology categories in panel (C). (F) Upper pie chart: number of kinetoplastid mitochondrial OGs shared with mitochondrial proteins from human,^76^ *S. cerevisiae*^84^ or *A. thaliana*.^77^ Lower pie chart: classification of the remaining 1,074 OGs based on the presence of PFAM domains and annotations derived from literature, Gene Ontology (GO), pathway databases, and TriTrypDB.^65^ (G) Heatmaps for cobalamin-associated enzymes (left) and GDP–fucose biosynthesis (right) showing mitochondrial genes present (blue) or absent (gray) in *T. brucei* and *L. tarentolae*. Asterisks denote cytosolic proteins; all other genes correspond to mitochondrial proteins in humans and either *T. brucei* or *L. tarentolae*. *Leptomonas: Leptomonas pyrrhocoris*.

**Key resources table:** 

REAGENT or RESOURCE	SOURCE	IDENTIFIER
Antibodies		
Anti-RESC1/2, rabbit polyclonal	In-house	N/A
Anti-KRET1, mouse monoclonal	In-house	N/A
Anti-TbATOM40, rabbit polyclonal	This study	N/A
Anti-LtATOM40, rabbit polyclonal	This study	N/A
Anti-biotin, HRP-linked antibody	Cell Signaling	7075
Anti-β-tubulin, clone KMX-1, mouse	MilliporeSigma	MAB3408
THE^™^ GFP Antibody, pAb, rabbit	GenScript	A01704100
StrepMAB-Immo, mouse	IBA Lifesciences	2-1517-001
F(ab’)2-Goat anti-Mouse IgG (H+L) Cross-Adsorbed Secondary Antibody, Alexa Fluor^™^ 647	Thermo Fisher Scientific	A21237
IBA LifeSciences Strep-TactinXT conjugate DY-488	IBA Lifesciences	21562050
Bacterial and virus strains		
		
Biological samples		
		
Chemicals, peptides, and recombinant proteins		
Brain Heart Infusion (BHI) broth	MilliporeSigma	53286
Hemin	MilliporeSigma	16009-13-5
MitoTracker Red CMXRos	Thermo Fisher Scientific	M7512
OptiPrep	Cosmo Bio USA Inc.	AXS1114542
cOmplete, Mini, EDTA-free Protease Inhibitor Cocktail	Roche	4693159001
DNase I	MilliporeSigma	11284932001
Hoechst 33342	Thermo Fisher Scientific	H3570
UltraPure SDS Solution, 10%	Thermo Fisher Scientific	15553-035
Triethylammonium bicarbonate buffer, 1 M	MilliporeSigma	18597-500ML
Magnesium chloride solution, BioUltra, Molecular Biology, 1 M	MilliporeSigma	63069-100ML
Aprotinin	MilliporeSigma	A6106-1MG
Leupeptin	Roche	11017101001
Phenylmethanesulfonyl fluoride solution, 0.1 M in ethanol	MilliporeSigma	93482-50ML-F
Benzonase nuclease	Thomas Scientific	E1014-25KU
DTT (Dithiothreitol), No-Weigh Format	Thermo Fisher Scientific	A39255
Iodoacetamide	MilliporeSigma	A3221-10VL
Formic acid	MilliporeSigma	5330020050
Acetonitrile	J.T.Baker	9829-03
Phosphoric acid	LabChem	LC186401
Trifluoroacetic acid	MilliporeSigma	302031-10X1ML
1-Propanol	MilliporeSigma	279544-1L
Sequencing Grade Modified Trypsin	Promega	V5113
Lysyl Endopeptidase R, Mass Spectrometry Grade (Lys-C)	FUJIFILM Wako	129-02541
Methanol	Honeywell	34966-4X4L
Proteinase K	MilliporeSigma	P55681ML
Critical commercial assays		
Pierce BCA Protein Assay Kits	Thermo Fisher Scientific	23225
S-Trap Mini Column	PROTIFI	C02-mini-40
S-Trap Micro Column	PROTIFI	C02-micro-40
Sep-Pak tC18 96-well Plate, 40 mg Sorbent per Well	Waters	186002320
Empore C18 Extraction Disks	CSD Analytical	76333-132
Evotip Pure, 96 tips	Evosep	EV2011
Human T cell Nucleofector Kit	Lonza	VVPA-1002
QuadroMACS Starting Kit (LS)	Miltenyi	130-091-051
Endurance Column, 30 SPD	Evosep	EV1106
Stainless Steel Emitters	Evosep	EV1086
96-Well Microplate Flat Bottom Clear	VWR	82050-760
PepSep C18 25cm × 75 μm, 1.5 μm	Bruker Daltonics	1893484
Aurora Elite 15×75 CSI C18 UHPLC column	IonOpticks	AUR4-15075C18-CSI
UNIPLATE microplates	Whatman	7701-5200
Deposited data		
Mass spectra and the protein sequence databases used for searches	MassIVE	MSV00010185
Fluorescent and electron microscopy data and uncropped gel and blot images	Mendeley Data	https://doi.org/10.17632/2m586kg4z7.1
Experimental models: Cell lines		
*Trypanosoma brucei*: POMP10-eGFP-TS	This study	N/A
*Trypanosoma brucei*: Tb927.11.2190-CTS	This study	N/A
*Trypanosoma brucei*: Tb927.10.3120-CTS	This study	N/A
*Trypanosoma brucei*: Tb927.10.660-CTS	This study	N/A
*Trypanosoma brucei*: Tb927.9.9970-CTS	This study	N/A
*Trypanosoma brucei*: Tb927.8.600-CTS	This study	N/A
*Trypanosoma brucei*: Tb927.8.1450-CTS	This study	N/A
*Trypanosoma brucei*: Tb927.11.7400-CTS	This study	N/A
*Leishmania tarentolae*: pX-POMP10-eGFP-TS	This study	N/A
*Leishmania tarentolae*: pX-LtaP06.0560-CTS	This study	N/A
*Leishmania tarentolae*: pX-LtaP28.1350-CTS	This study	N/A
*Leishmania tarentolae*: pX-LtaP26.2070-CTS	This study	N/A
*Leishmania tarentolae*: pX-LtaP08.0290-CTS	This study	N/A
*Leishmania tarentolae*: pX-LtaP21.1390-CTS	This study	N/A
*Leishmania tarentolae*: pX-LtaP33.0490-CTS	This study	N/A
*Leishmania tarentolae*: pX-LtaP29.2070-CTS	This study	N/A
*Leishmania tarentolae*: pX-LtaP32.1580-CTS	This study	N/A
*Leishmania tarentolae*: pX-LtaP06.0830-CTS	This study	N/A
*Leishmania tarentolae*: pX-LtaP14.1240-CTS	This study	N/A
*Leishmania tarentolae*: pX-LtaP30.1210-CTS	This study	N/A
*Leishmania tarentolae*: pX-LtaP08.0200-CTS	This study	N/A
Experimental models: Organisms/strains		
*Trypanosoma brucei* Lister 427 procyclic form	Larry Simpson, UCLA	N/A
*Trypanosoma brucei* Lister 427 bloodstream form	Larry Simpson, UCLA	N/A
*Leishmania tarentolae* (UC) strain	Larry Simpson, UCLA	N/A
Oligonucleotides		
Table S11		
Recombinant DNA		
Plasmid: pUC57-eGFP-TS	This study	N/A
Plasmid: pUC57-CTS	This study	N/A
Plasmid: pXG-POMP10-eGFP-TS	This study	N/A
Plasmid: pXG-LtaP26.2070-CTS	This study	N/A
Plasmid: pXG-LtaP08.0290-CTS	This study	N/A
Plasmid: pXG-LtaP29.2070-CTS	This study	N/A
Plasmid: pXG-LtaP06.0560-CTS	This study	N/A
Plasmid: pXG-LtaP28.1350-CTS	This study	N/A
Plasmid: pXG-LtaP14.1240-CTS	This study	N/A
Plasmid: pXG-LtaP06.0830-CTS	This study	N/A
Plasmid: pXG-LtaP30.1210-CTS	This study	N/A
Plasmid: pXG-LtaP21.1390-CTS	This study	N/A
Plasmid: pXG-LtaP32.1580-CTS	This study	N/A
Plasmid: pXG-LtaP33.0490-CTS	This study	N/A
Plasmid: pXG-LtaP08.0200-CTS	This study	N/A
Software and algorithms		
Spectronaut (version 19)	Biognosys	biognosys.com/software/spectronaut/
HMMER (version 3.1)	Potter et al.^88^	hmmer.org
OrthoFinder (version 2.5.5)	Emms et al.^100^	github.com/davidemms/OrthoFinder/releases
BLAST+ (version 2.2.30)	Camacho et al.^93^	ftp.ncbi.nlm.nih.gov/blast/executables/blast+/2.2.30/
DeepLoc2.1	Odum et al.^42^	services.healthtech.dtu.dk
GSEA	Subramanian et al. ^58^	www.gsea-msigdb.org
abSENSE-HMM, OrthoGroups, Phylogenetic tree	Chen et al. Cell	github.com/michaelzhuchen/mito-evolution
Other		
Mito-TOL website	This study	www.mitocarta.org
TriTrypDB	Shanmugasundram et al.^65^	tritrypdb.org
PFAM	Potter et al.^88^	www.ebi.ac.uk/interpro/download/pfam
kDNA edited sequences	Simpson et al.^87^	www.lifesci.ucla.edu/RNA/trypanosome/database.html
TrypTag	Billington et al.^11^	Table S3
HyperLOPIT	Moloney et al.^12^	Table S9, column tagm.map.localisation.prediction
Panigrahi mitoproteome	Panigrahi et al.^14^	Tables 1, 2, S5 and S6
Mitochondrial respiratome	Acestor et al.^18^	Tables S1 and S3
Mitochondrial importome	Peikert et al.^21^	Supplemental Data 1
Mitochondrial outer membrane proteome	Niemann et al.^24^	Table II and III
Tasbihi mitoproteome	Tasbihi et al.^23^	Table S1
Human MitoCarta3.0	Rath et al.^76^	www.broadinstitute.org/mitocarta
TbMitoCarta	This study	N/A
LtMitoCarta	This study	N/A
