# Supplementary material for: Lineage-specific innovations expand mitochondrial proteomes in kinetoplastids
Source: Mol Cell. Author manuscript; Available in PMC 2026 Nov 2. (PMC13725907; doi:10.1016/j.molcel.2026.08.032)
Supplement: Supplementary file 1 [file NIHMS2214060-supplement-SuppMaterials.pdf]

## SUPPLEMENTAL INFORMATION

# Lineage-specific innovations expand mitochondrial proteomes in kinetoplastids

Andres F. Vacas, Md Solayman, Sarah E. Calvo, Khanh Nguyen, Cameron Genxuan Lian, Keith D. Rivera, Michael Z. Chen, MitoCarta Tree of Life Consortium, Namrata D. Udeshi, Vamsi K. Mootha, Ruslan Aphasizhev, and Inna Aphasizheva

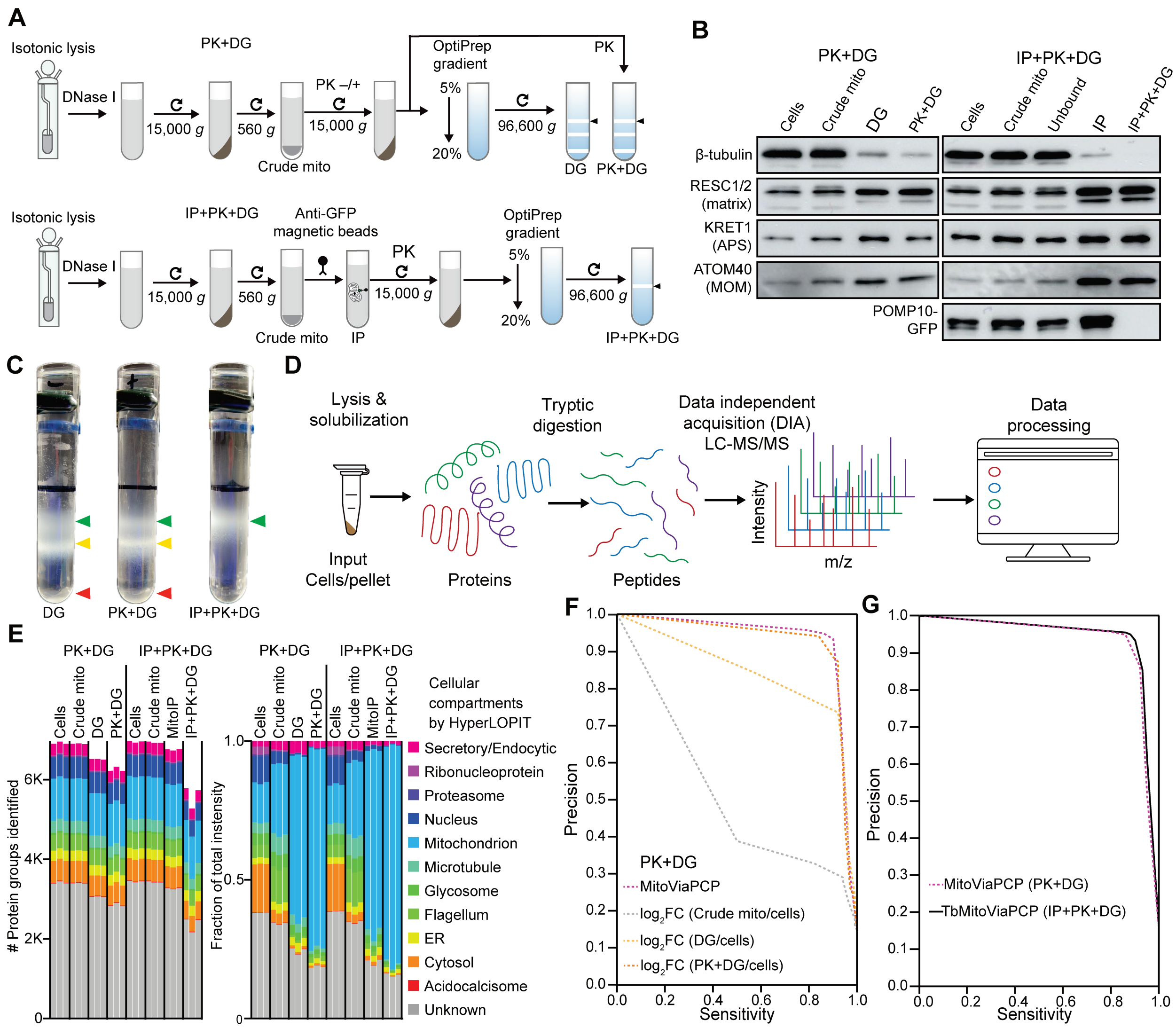


**Figure S1. Immunoblotting and DIA-MS analyses of mitochondrial purification workflows in *T. brucei*, related to Figure 1.**

(A) Schematics of *T. brucei* mitochondrial purification workflows: OptiPrep density-gradient centrifugation following proteinase K treatment (PK+DG) or immunoprecipitation followed by proteinase K treatment and OptiPrep density-gradient centrifugation (IP+PK+DG).
(B) Immunoblots of fractions across mitochondrial isolation from *T. brucei.* APS, kinetoplast antipodal sites; MOM, mitochondrial outer membrane.
(C) Photographs of OptiPrep density gradients. Bands indicated by arrowheads were analyzed by LC–MS/MS. A green arrowhead indicates the mitochondria-enriched band; cytoskeleton- and flagella-containing zones are indicated by yellow and red arrowheads, respectively.
(D) Workflow for data-independent acquisition mass spectrometry analysis.
(E) Bar plots showing the number of protein groups identified and the fraction of total MS intensity across purification steps. Proteins are colored according to their predicted subcellular compartment from the HyperLOPIT TbPCF dataset.
(F) Sensitivity and precision curves for fractions of increasing purity from the PK+DG workflow ($log_2$ fold-change using a single replicate) compared with protein correlation profiling (PCP).
(G) Sensitivity and precision curves comparing PCP results from IP+PK+DG and PK+DG workflows, showing moderate improvement with inclusion of the immunoprecipitation step.

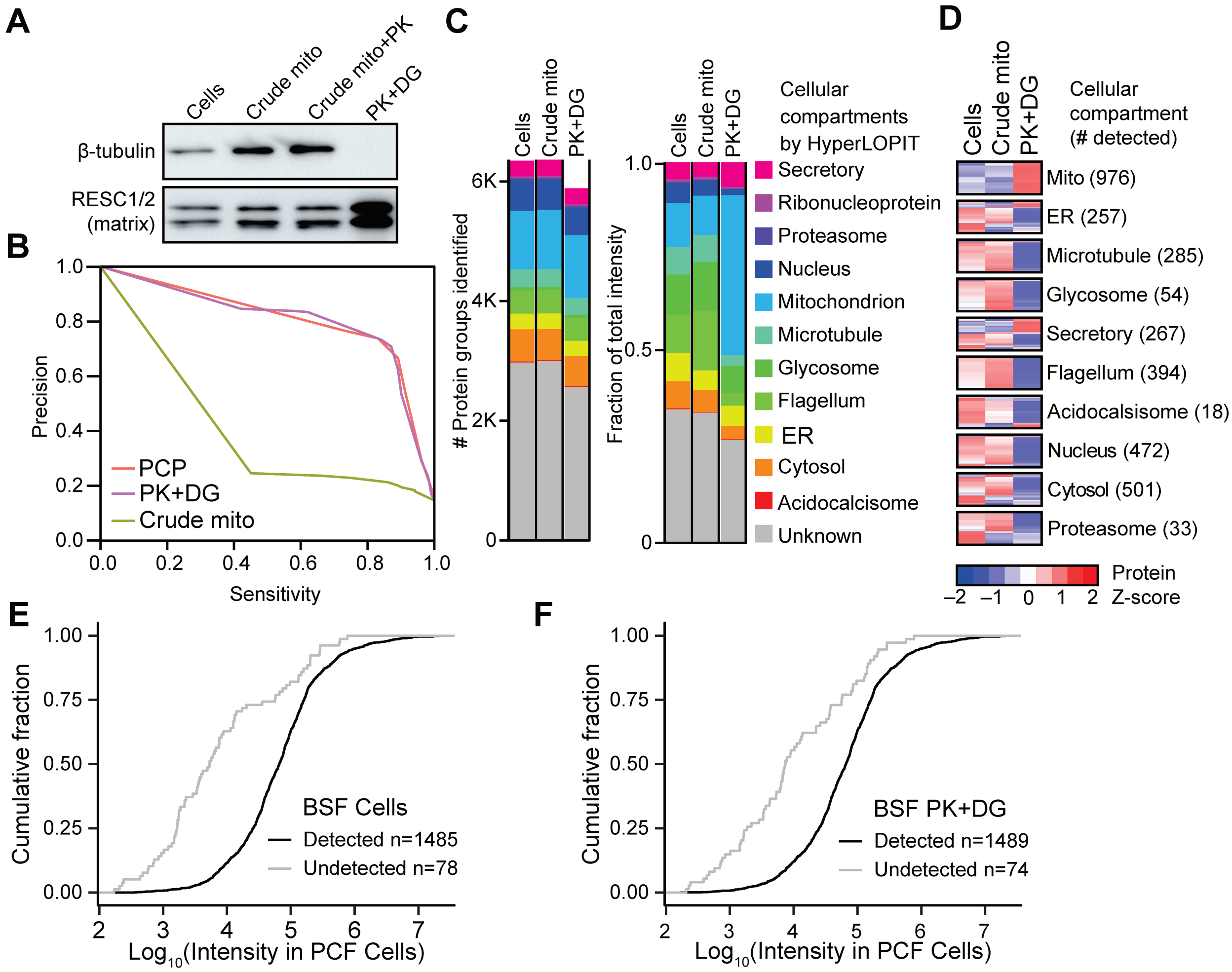


**Figure S2. DIA-MS analysis of mitochondrial purification workflow in bloodstream *T. brucei*, related to Figure 2.**

(A) Immunoblots of fractions across mitochondrial isolation from *T. brucei* bloodstream form.
(B) Sensitivity–precision curves for crude mitochondrial (crude mito), PK+DG, and PCP samples. The $\log_2$(fraction/whole-cell) ratios from a single replicate are plotted.
(C) Bar plots showing the number of protein groups identified by DIA-MS across purification fractions (left) and their corresponding fractions of total MS signal intensity (right). Protein groups are colored according to the predicted subcellular compartment of the first protein in each group, based on TbPCF HyperLOPIT annotations.
(D) Heatmap showing relative protein abundances quantified across three sequential purification steps (one replicate each). Each row represents one protein group, separated by predicted subcellular localization from TbPCF HyperLOPIT; the number of protein groups per compartment is indicated (limited to protein groups detected in all three samples).
(E) Empirical cumulative distribution function (eCDF) plot showing protein abundance in TbPCF cells for mitochondrial proteins (TbMitoViaPCP) that are detected or undetected in any of the three replicates of BSF cells profiling. The X-axis shows the mean $\log_{10}$(normalized intensity) of three replicates in TbPCF cells from the Figure 1 dataset.
(F) Empirical cumulative distribution function (eCDF) plot showing protein abundance in PCF cells for mitochondrial proteins (TbMitoViaPCP) that are detected or undetected in the single replicate of mitochondria enriched from BSF cells (PK+DG). The X-axis shows the mean $\log_{10}$(normalized intensity) of three replicates in PCF cells from the Figure 1 dataset.

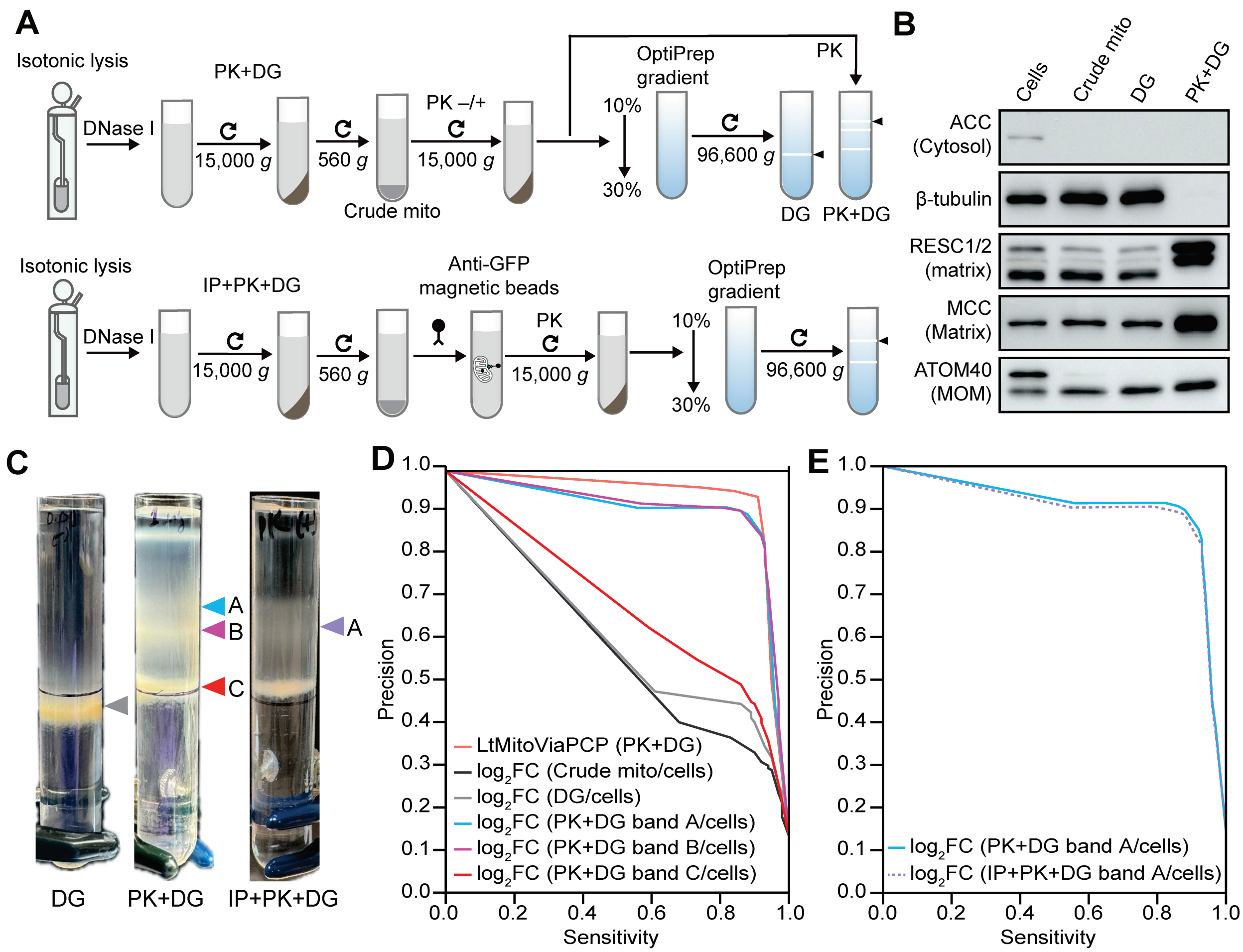


**Figure S3. Immunoblotting and DIA-MS analyses of mitochondrial purification workflows in *L. tarentolae*, related to Figure 3.**

(A) Schematics of *L. tarentolae* mitochondrial purification workflows: OptiPrep density-gradient centrifugation following proteinase K treatment (PK+DG) or immunoprecipitation followed by proteinase K treatment and OptiPrep density-gradient centrifugation (IP+PK+DG).
(B) Immunoblots of fractions collected along the PK+DG workflow for mitochondrial isolation from *L. tarentolae*. ACC, acetyl-CoA carboxylase; MCC, 3-methylcrotonyl-CoA carboxylase.
(C) Photographs of OptiPrep density gradients: mock (DG; left), PK-treated (PK+DG; middle), and immunoprecipitated and PK-treated (IP+PK+DG; right). Bands indicated by arrowheads were analyzed by LC-MS/MS to identify the mitochondria-enriched zone (Band A and B). The band containing cytoskeletal contaminants is also shown (Band C).
(D) Sensitivity–precision curves for samples obtained using the DG and PK+DG workflows. Band A shows moderate improvement over Band B.
(E) Sensitivity–precision curves from protein correlation profiling (PCP) showing a modest performance advantage of the PK+DG workflow over the IP+PK+DG workflow.

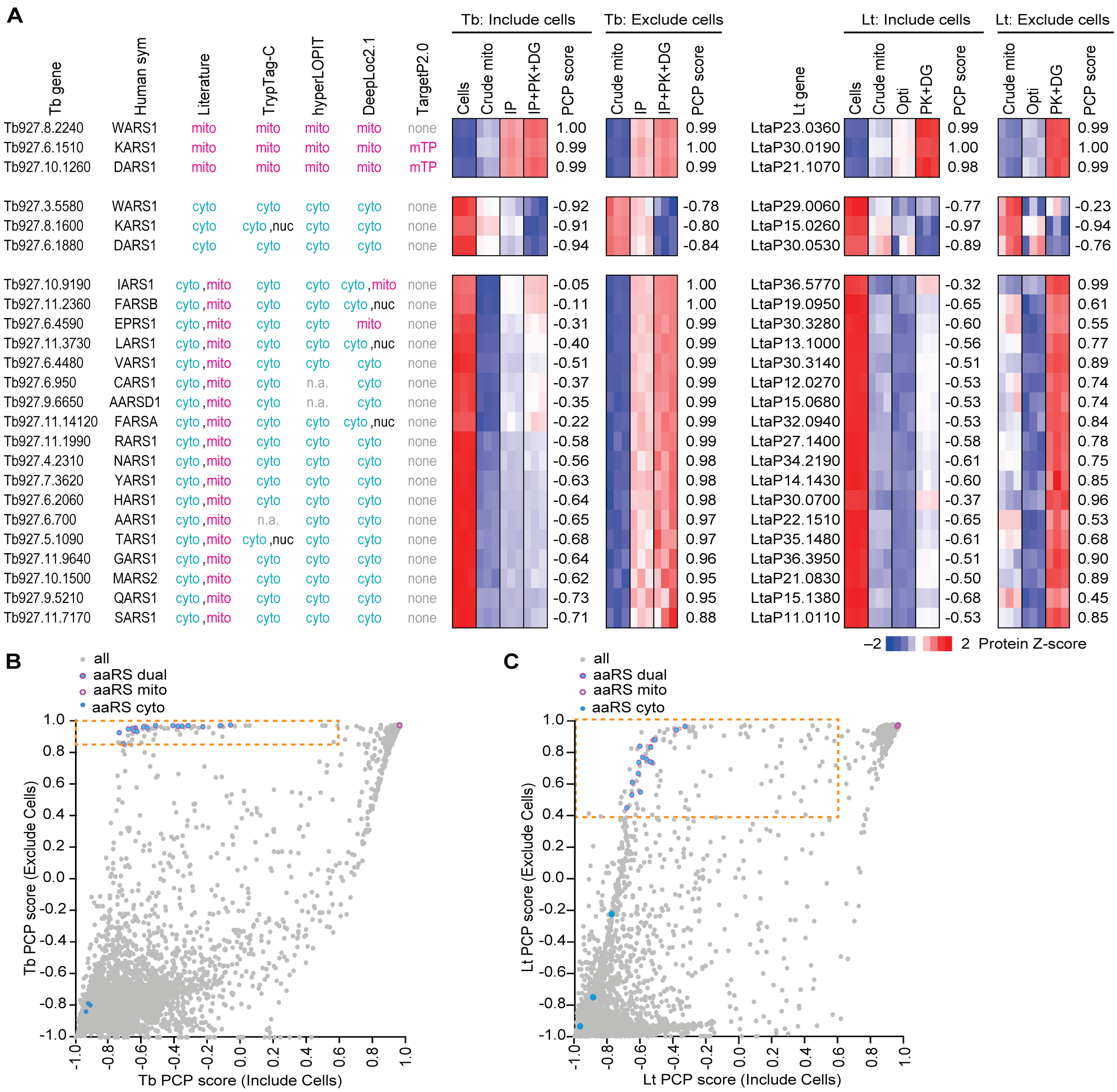


**Figure S4. Prediction of dual-targeted mitochondrial proteins in *T. brucei* and *L. tarentolae*.**

(A) Heatmap of fractionation profiles and PCP scores for aminoacyl-tRNA synthetases (aaRSs) with known mitochondrial, cytosolic, or dual localization. Heatmaps and PCP scores are shown for analyses that include all fractions and for analyses recalculated after the removal of whole-cell fractions. Annotations include human ortholog symbols and localization assignments based on literature, TrypTag, HyperLOPIT TbPCF, DeepLoc 2.1, and TargetP 2.0.
(B) Scatter plot of *T. brucei* proteins comparing PCP scores calculated with (X-axis) or without (Y-axis) whole-cell fractions. All proteins are shown in gray. The aaRSs are highlighted: cytosolic (cyan), mitochondrial (open pink circles), and dual-localized (pink circles with cyan centers). The dashed orange rectangle indicates the region enriched for dual-targeted candidates (PCP > 0.6 and PCP without whole-cell fractions > 0.85).
(C) Same analysis as in (**B**) for *L. tarentolae*. The dashed orange rectangle indicates the corresponding region for candidate dual-targeted proteins (PCP > 0.6 and PCP without whole-cell fractions > 0.4).
